# A comparison of methods for multiple degree of freedom testing in repeated measures RNA-sequencing experiments

**DOI:** 10.1186/s12874-022-01615-8

**Published:** 2022-05-28

**Authors:** Elizabeth A. Wynn, Brian E. Vestal, Tasha E. Fingerlin, Camille M. Moore

**Affiliations:** 1 Department of Biostatistics and Informatics, University of Colorado, Anschutz Medical Campus, Aurora, CO USA; 2 Center for Genes, Environment and Health, National Jewish Health, 1400 Jackson St, Denver, 80206 CO USA

**Keywords:** RNA-seq, Correlated data, Multiple DF testing

## Abstract

**Background:**

As the cost of RNA-sequencing decreases, complex study designs, including paired, longitudinal, and other correlated designs, become increasingly feasible. These studies often include multiple hypotheses and thus multiple degree of freedom tests, or tests that evaluate multiple hypotheses jointly, are often useful for filtering the gene list to a set of interesting features for further exploration while controlling the false discovery rate. Though there are several methods which have been proposed for analyzing correlated RNA-sequencing data, there has been little research evaluating and comparing the performance of multiple degree of freedom tests across methods.

**Methods:**

We evaluated 11 different methods for modelling correlated RNA-sequencing data by performing a simulation study to compare the false discovery rate, power, and model convergence rate across several hypothesis tests and sample size scenarios. We also applied each method to a real longitudinal RNA-sequencing dataset.

**Results:**

Linear mixed modelling using transformed data had the best false discovery rate control while maintaining relatively high power. However, this method had high model non-convergence, particularly at small sample sizes. No method had high power at the lowest sample size. We found a mix of conservative and anti-conservative behavior across the other methods, which was influenced by the sample size and the hypothesis being evaluated. The patterns observed in the simulation study were largely replicated in the analysis of a longitudinal study including data from intensive care unit patients experiencing cardiogenic or septic shock.

**Conclusions:**

Multiple degree of freedom testing is a valuable tool in longitudinal and other correlated RNA-sequencing experiments. Of the methods that we investigated, linear mixed modelling had the best overall combination of power and false discovery rate control. Other methods may also be appropriate in some scenarios.

**Supplementary Information:**

The online version contains supplementary material available at (10.1186/s12874-022-01615-8).

## Background

RNA-sequencing (RNA-seq) technology has revolutionized how we study and understand the underlying pathobiology of disease. Recently, declining sequencing costs have allowed for more complex investigations, including correlated and longitudinal study designs. In particular, longitudinal designs have become increasingly popular, as they allow researchers to understand the dynamics of gene expression across time and how these dynamics differ between groups of subjects. However, complex study designs demand more sophisticated analysis methods. As with single timepoint designs, careful pre-processing of longitudinal RNA-seq data is still necessary prior to analysis to remove artifacts produced during sequencing [1, 2]. Following pre-processing, distributional and computational considerations are necessary to model overdispersed count data on 10,000-20,000 genes. Additionally, analysis methods for longitudinal study designs must also account for the correlation induced by repeated measures, which is often achieved with random effects or modeling of the error covariance structure. To be most applicable to these complex study designs, analysis approaches should allow for flexible modeling, including the ability to adjust for potential confounders and subject demographics.

In longitudinal RNA-seq studies, researchers are often interested in multiple hypotheses. For example, many longitudinal RNA-seq studies include repeated measures from each subject over time, with subjects coming from multiple treatment groups. This allows for the investigation of between-subject comparisons, such as a test for differences in gene expression between treatment groups at a particular timepoint; within-subject comparisons, such as a test for differences in gene expression across two timepoints in a single treatment group; or interaction effects to compare changes over time between groups. Furthermore, studies with more than two timepoints per subject might involve multiple comparisons across different timepoints in order to characterize how gene expression changes across time.

In the situation where there are multiple hypotheses to be tested for each gene, the ability to perform an omnibus test, or a test where multiple hypotheses are evaluated, is valuable for controlling false discovery rates. For example, in a study with multiple timepoints per subject in which time is treated categorically, a researcher might wish to compile a list of genes that change over time for further investigation. In such a situation, one could perform a series of hypothesis tests to identify the differentially expressed genes (DEGs) between each pair of timepoints and perform a multiple testing correction to each hypothesis test individually to control the false discovery rate to 5%, for example. However, because each hypothesis test may produce different false positive genes, when lists of significant genes are aggregated across multiple hypotheses, the percentage of false positives in the aggregated list will be greater than 5% without additional adjustment [3]. Thus, performing an omnibus test for multiple hypotheses is useful in false discovery rate control. These types of tests are often referred to as multiple degree of freedom (DF) tests because the hypothesis for these tests involve multiple degrees of freedom as opposed to the single degree of freedom required for hypothesis testing of a single covariate or effect.

Several different methods have been proposed for the analysis of longitudinal RNA-seq data. Popular analysis packages such as edgeR [4, 5] and DESeq2 [6] are often appealing to researchers because they allow for flexible modelling in a generalized linear modelling (GLM) framework. However, these packages do not allow for random effects or covariance structures to properly accommodate correlated data. Despite this limitation, these packages are sometimes used to analyze correlated data, either by treating each subject/cluster as a fixed effect under a regression framework, or by ignoring the correlation altogether and treating correlated samples as independent. It is well established that ignoring correlation can lead to bias in standard error estimation which can influence the results of statistical tests [7]. Alternatively, treating each subject/cluster as a fixed effect may result in inflated false positive rates due to over-fitting [8]. Additionally, when coefficients for each subject/cluster are included in the model, other subject-level effects, such as group differences, are not estimable.

The limma [9] package, another popular analysis tool for RNA-seq data, includes the capability to account for correlation between related samples using a method in which a common correlation value estimated across all genes is incorporated into the model for each gene [10]. However, this method assumes that the correlation between samples is the same for all genes. This is a strong assumption that may not be true in practice.

Recently, several methods have been proposed for longitudinal and other correlated RNA-seq studies. These methods generally use random effects or covariance structures to account for the correlation in the data while also considering the unique characteristics of RNA-seq data such as overdispersion. Many methods developed for correlated RNA-seq data are limited by the fact that they do not allow for multiple treatment groups or additional covariates (e.g. PLNseq [11], multiDE [12]), can only be used for paired data (e.g. baySeq [13, 14], PairedFB [15]), or can only perform single DF tests (e.g. MCMSeq [16], ShrinkBayes [17]).

Some researchers have proposed employing standard statistical models typically used for longitudinal and correlated data outside of the context of RNA-seq data, as these well-developed modeling frameworks allow for flexible modeling and hypothesis testing [18–20]. In applying these methods to RNA-seq data, considerations still must be made to account for the non-normality of the data, for example, by choosing a repeated measures model with an underlying distribution for overdispersed counts.

Tsonaka & Spitali [20] investigated the use of negative binomial mixed models (NBMM) for RNA-seq data using an adaptive Gaussian quadrature method to estimate parameters and found that this method was relatively unbiased and exhibited type 1 error (T1E) and false discovery rate (FDR) control. Similarly, Zhang et al. [21] used NBMM to analyze correlated microbiome data, which are also overdispersed counts, but used an iterative weighted least squares (pseudo-likelihood) approach for parameter estimation. They demonstrated the utility of the method through both simulation study and application to mouse gut microbiome data. Rather than using the negative binomial distribution, Park et al. [19] investigated the use of generalized estimating equation (GEE) models using a Poisson distribution with an extra scale parameter to account for overdispersion. They found that these models identified more DEGs than edgeR, DESeq or limma, though they did not explore whether this was driven by high false positive rates.

Instead of directly modeling counts, another approach is to normalize the data and then utilize models that assume a normal distribution. The package rmRNAseq [18] utilizes the voom normalization method on log-transformed counts and then models the transformed data using a linear model with a continuous auto-regressive structure to account for the correlation in the data. Vestal et al. [16] tested a similar method by using a variance stabilizing transformation (VST) on raw RNA-seq counts and then fitting linear mixed models (LMMs) to the transformed data. They found that this method performed similarly to their hierarchical Bayesian MCMSeq method in terms of T1E and FDR control, but many models failed to converge in small sample size situations.

All of the methods outlined above allow for multiple DF hypothesis testing. However, there has been little research evaluating and comparing the performance of multiple DF tests across these methods. Some studies have evaluated the use of multiple DF tests for a single method or in comparison to DESeq2 and edgeR, which do not account for correlation, rather than methods that account for correlation [18, 20]. Others have compared multiple correlated data approaches but only for single DF hypothesis tests [16]. As complex study designs become more common in correlated RNA-seq designs, multiple DF hypothesis testing is important for identifying interesting genes for downstream analysis without increasing the FDR.

In this paper, we compare the performance of several methods for analyzing correlated RNA-seq count data with particular emphasis on multiple DF test performance within each method. First, we investigate model performance through a simulation study. Each method is also applied to RNA-seq data collected from septic shock and cardiogenic shock patients over multiple timepoints following admission to the intensive care unit (ICU). Finally, we provide recommendations as to which models are most appropriate under various circumstances.

## Methods

### Analysis methods compared

We compared methods which have been proposed for correlated RNA-seq experiments and that allow for multiple treatment groups, covariates and/or timepoints, and can be used to perform multiple DF tests. We describe the selected methods below. Additional information on each method is available in Supplementary Materials Section 1.

#### Standard RNA-seq analysis tools

Standard RNA-seq analysis tools generally use a linear modelling framework with transformed data, or a generalized linear model (GLM) framework, assuming a negative binomial distribution. In studies with correlated designs, these methods can be implemented with the caveat that the model assumptions, such as the independence of observations, will not be met, or adjustments can be made to attempt to account for the correlation of the data. In this study, we tested three of the methods from the most popular RNA-seq analysis packages: limma, edgeR, and DESeq2.

The R package limma was originally created for the analysis of microarray expression data, which are approximately normally distributed [9]. limma employs linear models to test for differential expression using an empirical Bayes approach to share information across genes. This methodology has been extended to RNA-seq data by applying the “voom” transformation to RNA-seq counts [22, 23]. First, RNA-seq counts are normalized using the log counts per million (log-CPM) transformation. A mean-variance relationship is then estimated, and from this relationship, a predicted variance is calculated for each log-CPM value, which is then incorporated into a linear model as an inverse weight. The duplicateCorrelation function within the package can be used to estimate correlation values for each subject which are then incorporated in the linear model. However, only one correlation is computed for all genes.

The edgeR and DESeq2 packages both employ a negative binomial GLM framework to address overdispersion [4–6]. Both methods use empirical Bayes procedures to estimate variability, effectively borrowing information across genes to inform the estimation. Both methods also include offset terms in their models to account for differences in library size between samples, though edgeR uses the trimmed mean of M-values (TMM) method [4], while DESeq2 uses the median ratio method [24]. These packages do not include methods to account for correlation between samples.

#### Generalized estimating equations

Generalized estimating equations (GEE) are a semi-parametric extension of GLM that can account for correlation between observations [25]. This method uses a working correlation structure to model the association between measurements within a subject. The covariance matrix of the estimated regression coefficients is typically estimated using robust (sandwich) estimators so that the estimates are robust to misspecification of the working correlation structure. In this analysis, we modelled the data using a Poisson distribution with an extra scale parameter in the variance to account for overdispersion, and an exchangeable working correlation structure.

One drawback to GEE models is that sandwich estimators have poor performance at small sample sizes. To address this issue, we used the small sample size adjustment proposed by Wang and Long [26], which utilizes information from all subjects to calculate the covariance for each individual subject and also uses an additional adjustment to correct for bias.

#### Negative binomial mixed models

Generalized linear mixed models (GLMM) are an extension of GLMs that use random effects to account for correlation. Similar to the methods implemented in edgeR and DESeq2, in using the GLMM framework, the gene expression for each gene can be modeled using a negative binomial distribution, which accounts for the overdispersion.

When using negative binomial mixed modelling (NBMM), parameter estimation can be analytically complex and there are multiple approaches that can be used. We consider two maximum likelihood estimation approaches, Laplace (NBMM-LP) and adaptive Gaussian quadrature (NBMM-AGQ) as well as the pseudolikelihood approach (NBMM-PL).

#### rmRNAseq and linear mixed models

The rmRNAseq package employs a method similar to the limma+voom method in which the data are first transformed using the voom approach and then a linear model is fit for each gene using the transformed data. However, within the rmRNAseq framework, models are fit using a continuous autoregressive correlation structure to account for correlation in the data.

A similar approach is to use linear mixed modelling (LMM) with random effects to account for correlated data after applying a normalizing transformation. We test this approach using a variance stabilizing transformation (VST), as demonstrated in Vestal et al. [16].

### Implementation

We implemented each method using R (version 4.0.2). All analysis was carried out on a Linux high performance computing (HPC) cluster and parallel processing with 8 cores was used for all methods besides limma, DESeq2, and edgeR. Table 1 contains the specific packages used for each method and implementation details. Where possible, we used previously implemented R packages. In some cases, available R packages were missing important functionality, such as the capacity to account for offsets (geesvm for GEE small sample estimators). In these cases, custom R functions were built using the source code from the previously implemented R packages as a framework. Functions for implementing and summarizing results for methods in which no wrapper/summarization functions were available can be found in the corrRNASeq package, which is available at https://github.com/ewynn610/corrRNASeq.

**Table 1:** Analysis methods with their associated R packages and details concerning their implementation

Method	R-Package(s)	Multiple DF Test	Library Adjustment Offset	Details
DESeq2	DESeq2 [6]	LRT	DESeq2 size factors	Default settings used, correlation ignored.
DESeq2 ^∗^	DESeq2 [6]	LRT	DESeq2 size factors	Default settings used, subjects treated as fixed effects to account for correlation.
edgeR	edgeR [4]	LRT	TMM offset	Default settings used, correlation ignored.
edgeR ^∗^	edgeR [4]	LRT	TMM offset	Default settings used, subjects treated as fixed effects to account for correlation.
limma	limma [9, 23]	Moderated F-test	NA	Count data transformed using the voom function. The duplicateCorrelation function was used with subject id as a blocking factor to account for correlation.
GEE	Custom R Functions, geepack [27]	Wald *χ*^2^ Test	DESeq2 size factors	Models fit using exchangeable working correlation structure. For small sample estimators, custom functions were created by modifying code from the geesmv package [28] to make it compatible with geepack and enable the use of offsets.
LMM	lmerTest [29]	F-test	NA	Data transformed using the variance stabilizing transformation from the DESeq2 package.
NBMM-AGQ	GLMMadaptive [30]	LRT	DESeq2 size factors	Model fit using the mixed_model function with a negative binomial distribution. Default settings used for all other parameters.
NBMM-LP	glmmADMB [31]	LRT	DESeq2 size factors	Model fit using the glmmadmb function with a negative binomial distribution. Default settings used for all other parameters.
NBMM-PL	Custom R Function	LRT	DESeq2 size factors	Custom function was created drawing from the glmm.nb function in the NBZIMM package [21]. Function was created to be compatible with the lmerTest package [29] in order for Satterthwaite degrees of freedom to be calculated.
rmRNAseq	rmRNAseq [18]	Moderated F-statistic with bootstrapped *p*-values	NA	Model fit using the TC_CAR1 function with the default parameters.

Offsets to adjust for differences in library size were included in models for all except three methods (Table 1). The transformations used in limma, rmRNAseq and the LMM method accounted for differences in library size, so no additional adjustment was used.

The models using the edgeR and DESeq2 packages were fit in two ways. First, correlation was ignored and a model was fit with an intercept, time and group main effects, and an interaction term. Second, a fixed effect for subject was included in the model (edgeR ^∗^ and DESeq2 ^∗^). When including this extra fixed effect, the group term was not included in the model as it is inestimable.

Models were designated as non-converged if a maximum number of iterations were run without convergence during model fitting, models were found to be singular, or other errors prevented the model from fitting properly. All models that did not converge were discarded before further analysis.

#### Hypothesis testing

The packages used to implement each method in this analysis utilize different types of multiple DF tests. Table 1 shows the class of tests used for each method.

We used likelihood ratio tests (LRT) for the edgeR, DESeq2, NBMM-LP and NBMM-AGQ analyses. For all of these methods excluding edgeR, this required fitting two models for each test, a full model as well as a reduced model. The GLMMadaptive package used for fitting NBMM-LP models offers the option of using a multivariate Wald test instead of an LRT test. However Tsonaka & Spitali [20] found that in the context of correlated RNA-seq data, using LRTs resulted in lower T1E rate and FDR and thus we chose to use LRTs rather than multivariate Wald tests for these models. Additionally, Tsonaka & Spitali [20] proposed a bootstrap procedure for calculating *p*-values, particularly in small sample size situations. However, in running the example code provided with their publication, we found that it took about 2 hours to fit models and perform hypothesis testing for 10 genes with 1,000 bootstrap samples each. Because RNA-seq studies typically include 10,000-20,000 genes, this bootstrapping approach is likely not computationally feasible for most studies and we did not include it in our analysis.

Hypothesis testing for GEE was done using a Wald *χ*^2^ test as implemented by the esticon function in the doBy package [32]. F-tests were used for LMM and NBMM-PL and the Satterthwaite method was used to calculate denominator degrees of freedom [33, 34]. The limma and rmRNAseq packages both utilize the moderated *F*-statistic outlined by Smyth [35] for hypothesis testing. Under the limma framework, *p*-values are computed using an F-test with augmented degrees of freedom. The rmRNASeq package calculates *p*-values by building a distribution of null test statistics from data generated by a parametric bootstrap procedure and then computing the proportion of null statistics greater than or equal to the observed *F*-statistic.

### Simulation

#### Data generation

In order to evaluate and compare the testing characteristics of the previously described methods, we performed a simulation study. We used a two group design (e.g. treatment and control) with four observations per subject. A negative binomial distribution was used to simulate a matrix of counts **Y**. Let *Y*_*gij*_ be the expression level of gene *g* for the *i*th subject and *jth* observation, with *E*(*Y*_*gij*_)=*μ*_*gij*_. Further, let *α*_*g*_ be a dispersion parameter for gene *g* with $Var(Y_{gij})=\mu _{gij}+\alpha _{g}\mu ^{2}_{gij}$. Then

1 $$\begin{array}{*{20}l}  Y_{gij} &\sim& \mathcal{NB}(\mu_{gij}, \alpha_{g}) \end{array} $$

2 $$\begin{array}{*{20}l}  log(\mu_{gij}) &\!=& \!\beta_{g0}+\beta_{g1}X_{1i}+\beta_{g2}X_{2ij}+\beta_{g3}X_{3ij}+\beta_{g4}X_{4ij} \end{array} $$

3 $$\begin{array}{*{20}l} & &+\beta_{g5}X_{1i}X_{2ij} +\beta_{g6}X_{1i}X_{3ij}+\beta_{g7}X_{1i}X_{4ij}+b_{gi}  \\ b_{gi}&\sim& \mathcal{N}(0, \sigma^{2}_{g}) \end{array} $$

where *X*_1*i*_ is an indicator variable signifying whether the *i*th subject is in the treatment group or not, and *X*_2*ij*_, *X*_3*ij*_ and *X*_4*ij*_ are indicator variables representing whether observation *j* was taken at the 2nd, 3rd, or 4th timepoint respectively. Each *β*_*gk*_,*k*∈0,...,7 is a fixed effect regression coefficient specific to gene *g*. Finally, *b*_*gi*_ is the random intercept for gene *g* and subject *i* which is normally distributed with a mean of 0 and a variance of $\sigma ^{2}_{g}$.

Table 2 shows a summary of the simulation settings and multiple DF tests performed. We simulated 10 datasets for each simulation scenario. For each dataset we simulated 15,000 genes and then genes were filtered out if *N* samples had less than 1 count per million (CPM), where *N* was equal to the number of samples collected for a single group and timepoint. We simulated datasets to contain a mix of null and differentially expressed genes by changing the interaction coefficients for 20% of genes. In order to mimic real data, *β*_*g*0_,*α*_*g*_ and $\sigma ^{2}_{g}$, were drawn from an empirical distribution for triplets of mean CPM, dispersion, and random intercept variance observed across human samples in several real RNA-seq data sets with repeated measures [36, 37]. The fixed effect intercept parameter, *β*_*g*0_ was derived by scaling the randomly drawn CPM values to add up to one million and then multiplying each scaled value by a total library size of 25 million. Then, *β*_*g*0_ was set to the log of this value.

**Table 2:** Summary of simulated datasets

**Number of datasets**	10
**Number of genes per dataset**	∼ 15,000
**Sample sizes**	3, 5, and 10 per group
**Number of observation per subject**	4
**Model Parameters**	
*β*_*g*1_: Difference in log(expression) between treatment and control at baseline	0 (all genes)
*β*_*g*2_,*β*_*g*3_,*β*_*g*4_: Change in log(expression) over time in the control group	0 (all genes)
*β*_*g*5_,*β*_*g*6_,*β*_*g*7_: Difference in change in log(expression) over time between the treatment and control groups	0 (80% of genes), *β*_*g*5_=±1/3,*β*_*g*6_=±2/3,*β*_*g*7_=±1 (20% of genes)
$\beta _{g0}, \alpha _{g}, \sigma ^{2}_{gb}$	Drawn from an empirical distribution based on human samples in real RNA-seq data sets with repeated measures [36, 37]
**Significance tests**	
Between-subject	Are there differences in expression between the treatment and control at any of the time points? *H*_0_:*β*_*g*1_=*β*_*g*1_+*β*_*g*5_=*β*_*g*1_+*β*_*g*6_=*β*_*g*1_+*β*_*g*7_=0
Within-subject	Is there a change in gene expression between any timepoints for the treatment group? *H*_0_:*β*_*g*2_+*β*_*g*5_=*β*_*g*3_+*β*_*g*6_=*β*_*g*4_+*β*_*g*7_=(*β*_*g*2_+*β*_*g*5_)−(*β*_*g*3_+*β*_*g*6_)=(*β*_*g*2_+*β*_*g*5_)−(*β*_*g*4_+*β*_*g*7_)=(*β*_*g*3_+*β*_*g*6_)−(*β*_*g*4_+*β*_*g*7_)=0
Interaction	Are there any significant interaction effects? *β*_*g*5_=*β*_*g*6_=*β*_*g*7_=0
Global	Are there any significant model coefficients? *H*_0_:*β*_*g*1_=*β*_*g*2_=*β*_*g*3_=*β*_*g*4_=*β*_*g*5_=*β*_*g*6_=*β*_*g*7_=0

#### Simulation analysis

We analyzed simulated data using each method as described in the implementation section. Models for each gene were fit using fixed effects for group and time variables, which were both treated as categorical, as well as the interaction between group and time. A random intercept for each subject was included in models for methods in which random effects are possible. After the models were fit, the percentage of models that successfully converged for each method was calculated, and non-converged models were removed. Then the false discovery rate (FDR) and power were calculated for four different multiple DF tests: a between-subject test, a within-subject test, an interaction test, and a global test (Table 2). Power and FDR were calculated using Benjamini Hochberg adjusted *p*-values [38] and a significance threshold of 0.05 was used. For each simulation scenario, we averaged the statistics across 10 simulated datasets.

### Real data analysis

We applied the analysis methods previously outlined to a publicly available, longitudinal RNA-seq dataset of 96 whole blood samples from 32 patients experiencing circulatory shock who were admitted into the ICU (GEO Dataset: GSE131411). For each patient, three blood samples were collected: one within 16 hours after ICU admission, one 48 hours after admission, and one seven days after admission or at discharge. Subjects were categorized by whether they experienced septic shock (SS, *N*=21) or cardiogenic shock (CS, *N*=11). Further information on the study design and methods is available in Braga et al. [39].

#### Data pre-processing and model information

We downloaded the count table and study meta data from the GEO DataSets website. The data included 58,096 genes. We filtered out lowly expressed genes by removing genes that did not have greater than 1 CPM in at least 11 of the 96 samples (11 was the sample size in the smallest experimental group of interest), which reduced the total number of genes analyzed to 14,340.

The goal of our analysis was to investigate how the gene expression of shock patients changed over time and how these changes differed between patients with SS versus CS. To accomplish this, for each method we fit a model with fixed effects for the type of shock and timepoint (treated categorically) as well as the interaction between the two variables. A random intercept for each subject was included in models for methods in which random effects are possible. All models were fit as described in the implementation section. As with the simulation study, the percentage of models that failed to converge for each method was calculated and non-converged models were removed.

For each model, we ran four different multiple DF hypothesis tests: a between-subject test to assess if there was a difference in gene expression between the SS and CS groups at any timepoint, two within-subject tests to assess if there was a change in gene expression over time in the SS group or the CS group, and a test to assess if any of the interaction coefficients were significant. The Benjamini Hochberg method was used to adjust *p*-values for multiple comparisons and the DEGs for each method and test were identified using a 0.05 FDR threshold.

#### Hierarchical clustering and functional enrichment analysis

Because LMM exhibited comparatively good behavior in the simulation study, we used the results from this method to explore the patterns in the changes in gene expression over time in the SS and CS groups. All analysis was done for each group separately. First, we subset the data to include only genes that were significant in the multiple DF test for difference in gene expression at any timepoint in the SS group or CS group. For these genes, we computed the predicted gene expression (log scale) for each gene at each of the three timepoints for the group in question. We then constructed heatmaps for these genes, with genes clustered hierarchically using a correlation distance metric and a complete linkage clustering method. We visually inspected the heatmaps to decide where to cut each clustering tree to identify clusters that represented distinct profiles of change over time.

After clustering, we ran functional enrichment analysis on the genes in each cluster to better understand the functional role of genes with different expression profiles over time. Analysis was executed using the topGO package in R [40] using biological process biological process gene ontology (GO) annotations. The significance of the GO terms was assessed using a Fisher’s exact test with an FDR level of 0.05 as the threshold for significance. We further filtered the results to include only GO terms with at least 10 genes and > 10% overlap of the genes associated with each GO term and the genes in the cluster.

## Results

### Simulation results

#### Convergence

**Fig. 1:**
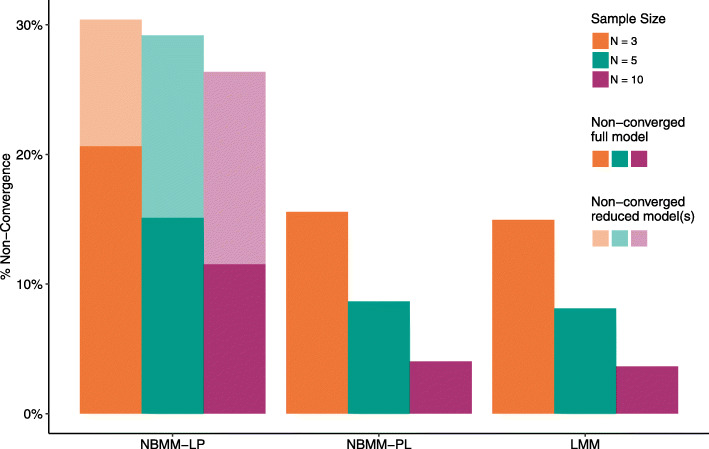
Percentage of non-converged models from selected methods. Methods in which less than 1% of models failed to converge are not included in the figure. For NBMM-LP, which uses a likelihood ratio test, the solid portion of the bar represents the proportion of models in which the full model did not converge and the transparent portion represents genes for which the reduced model for one or more tests failed to converge in which case results for those tests could not be obtained

Of the 11 methods evaluated, only 3 methods (NBMM-LP, NBMM-PL, and LMM) had average non-convergence rates above 0.1*%* for any of the sample sizes tested. Figure 1 shows the average percentage of models which did not converge across sample sizes for these methods. Because we used LRTs for NBMM-LP, for every gene a reduced model was fit for each of the four hypothesis tests. In some cases the full model converged but one or more of the reduced models failed to converge and thus the *p*-value for the corresponding hypothesis tests could not be calculated. The transparent portion of the bars in Fig. 1 represent cases in which the full model converged but one or more of the reduced models failed to converge.

NBMM-LP had the highest non-convergence rates at all sample sizes, even when only considering cases in which only the full model did not converge. At *N*=3 per group, about 21% of the full models did not converge and the reduced model(s) for an additional 10% of genes did not converge. Comparatively, at *N*=3 per group around 16% and 15% of models did not converge for NBMM-PL and LMM respectively. For all three methods, non-convergence rates decreased with increasing sample size, though the magnitude of the decrease was larger for NBMM-PL and LMM than for NBMM-LP. At *N*=10 per group, NBMM-PL and LMM both had non-convergence rates around 4% while NBMM-LP had a non-convergence rate of 11% with at least one reduced model failing to converge for an additional 15% of genes.

For all three methods and at all sample sizes, at least 90% of convergence failures were due to model singularities, with remaining non-converged models reaching model iteration limits or experiencing other errors which prevented the model from fitting properly. On average, the random intercept variance used to simulate the data was lower for genes that did not converge while the dispersion was generally higher (Supplementary Fig. 1). These results indicate that in some cases, model convergence issues may be due in part to low between-subject variation or high dispersion. However, there was substantial overlap in the random intercept and dispersion distributions between genes that did and did not converge, and many genes with high random intercept variance and low dispersion still failed to converge. In addition, the proportion of non-converged genes generally decreased only slightly (0.75%-1%) when using a higher expression filtering threshold of 5 CPM instead of 1 CPM, indicating that small expression values are also not completely responsible for model non-convergence (Supplementary Table 1).

#### Hypothesis testing

**Fig. 2:**
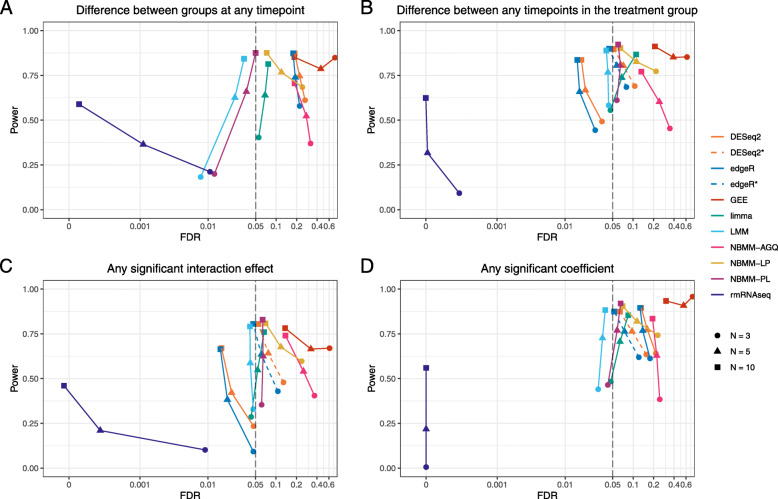
FDR versus power across different sample sizes for four tests of interest. FDR and power were calculated using a 0.05 FDR significance level and were averaged across 10 simulations for each method and sample size. Points that lie to the left of the dashed vertical line represent methods that have an observed FDR less than the nominal rate of 5%, while points to the right represent methods with FDR inflation. Points located in the bottom left-hand corner with an FDR and power of 0 represent instances in which no genes were found significant. A log scale is used on the x-axis to better differentiate between methods with close to nominal FDR

Figure 2 shows the relationship between FDR and power across different sample sizes for the four multiple DF tests of interest using a 0.05 FDR level. More detailed results are available in Supplementary Tables 2-4. The FDR for GEE, NBMM-AGQ, and NBMM-LP was higher than the nominal 0.05 level across all sample sizes for all tests. Other methods showed a mix of conservative and anti-conservative behavior. Across all tests, limma had an FDR close to the nominal rate for the smallest sample size (*N*=3 per group), but the FDR was increasingly inflated for the larger sample sizes. Conversely, DESeq2* and edgeR* had an inflated FDR at *N*=3 and *N*=5 per group, but at *N*=10 per group the rate was close to the nominal value. DESeq2 and edgeR (ignoring correlation) both had conservative FDR for the interaction and within-subject test, but showed inflated rates for the between-subject test and test for any significant coefficient. Across all of the tests, LMM was slightly conservative while NBMM-PL was slightly inflated except for the between-subject test, in which it was conservative. Finally, rmRNASeq had very conservative FDR values across all tests. For the majority of methods and tests, FDR approached the nominal rate (dashed line) and had increasing power with increasing sample size.

Of the methods that had FDR values which were conservative or close to the nominal rate across all sample sizes and conditions, LMM and NBMM-PL generally had the highest power. rmRNASeq, which showed conservative FDR values, had low power, particularly at the smaller sample sizes. For the within-subject test and the test for significant interaction effects in which edgeR and DESeq2 (ignoring correlation) exhibited conservative FDR values, both methods were less powered than LMM and NBMM-PL at all sample sizes. DESeq2* and edgeR*, which had close to nominal FDR values at *N*=10 per group, showed similar power to LMM and NBMM-PL at this sample size. Similarly, limma, which had close to nominal FDR at *N*=3 per group, had comparable power to LMM and NBMM-PL for most tests at this sample size and had more power than either method for the between-subject test.

At the smallest sample size, *N*=3 per group, no method that had conservative or close to nominal FDR had high power. For the within-subject test, LMM, NBMM-PL and limma had power values near 60% at *N*=3 per group, but no other tests showed power values this high for methods without severely inflated FDR. The power values at *N*=5 and *N*=10 per group were much stronger with LMM and NBMM-PL having power values near or above 80% for all tests at *N*=10 per group.

The distributions of the raw *p*-values from the null features in each simulated dataset are shown for each combination of method, test, and sample size in Supplementary Figs. 2-4. In general, we would expect these distributions to look fairly uniform. However, only LMM displays this behavior consistently. Some other methods, like NBMM-PL, limma at the smaller sample sizes, and DESeq2* and edgeR* at the larger sample sizes, are not too far off. Conversely, DESeq2, edgeR, GEE, and rmRNAseq show substantial skew. This suggests that the assumed distributions for the test statistics used in these methods is incorrect, and thus inference from these methods is likely compromised [41].

### Real data results

#### Run time

Table 3 shows the run time for each of the methods. The time to fit the full model and the total time (model fitting and hypothesis testing) are both shown for all methods except rmRNAseq, for which the model fitting and testing are carried out within one function and thus the run times cannot be uncoupled. NBMM-AGQ, NBMM-LP and both DESeq2 methods use an LRT which requires a full and reduced model to be fit for each hypothesis test, so for these methods hypothesis testing took a relatively large amount of time compared to the time to fit the full model. NBMM-LP had the longest total run time by far, taking over 24 hours to complete. The second highest run time was for rmRNAseq which took around 7 hours. Aside from these two methods, NBMM-AGQ (102 minutes), and NBMM-PL (65 minutes), all other methods ran in less than 30 minutes.

**Table 3:** Non-convergence rate, analysis run time, and number of DEGs for 4 hypothesis tests in the shock dataset. The run time for fitting the full model for each gene, as well as the total time to fit models and perform hypothesis testing is displayed. There were 14,340 genes in the dataset and genes were labelled as a DEG if the Benjamini Hochberg adjusted *p*-value was < 0.05. For NBMM-LP, the percentage of genes in which one or more reduced models failed to converge is shown in parentheses after the full model non-convergence rate

	DESeq2	DESeq2*	edgeR	edgeR*	GEE	limma	LMM	NBMM-AGQ	NBMM-LP	NBMM-PL	rmRNAseq
% Non-converged	0%	0%	0%	0%	0.04%	0%	0.31%	0%	4.33% (9.07%)	0.45%	0%
**Analysis Time (minutes)**											
Model Fit Time	1.14	6.44	0.61	5.8	11.01	2.16	8.91	24.83	326.74	62.72	-
Total Time (Model Fit & Hypothesis Testing)	6.05	28.49	0.72	6.76	18.19	2.2	11.76	104.78	1450.86	65.6	415.12
**Between Subject Test**											
SS vs. CS (Any timepoint)	5,323	-	5,179	-	4,034	3,801	3,009	3,303	3,633	3,351	3,461
**Within Subject Tests**											
Change over time - SS group	5,252	8,331	5,155	8,319	7,910	9,796	8,048	8,600	7,772	8,281	8,081
Change over time - CS group	128	1,381	123	1,313	872	638	1,003	1,836	1,280	1,229	168
**Interaction Test**											
Any Significant Interaction Effect	369	1,842	358	1,783	1,416	1,962	1,502	2,356	1,801	1,769	1,045

#### Model convergence

NBMM-LP had the largest percentage of non-converged models with 4.33% of the full model fits not converging (Table 3). An additional 9.07% of models did not converge for one or more reduced models used for LRTs, making the corresponding hypothesis test(s) incomputable. The non-convergence rate for the rest of the methods was less than 1%. This differed from the simulation results in which NBMM-PL and LMM had a non-convergence rate of around 4% at the largest sample size. The percentage of non-convergence for NBMM-LP was also smaller than for the largest sample size simulation scenario. This discrepancy is likely due in part to the large number of subjects in the shock dataset (32 total subjects; SS group: 21 subjects, CS group: 11 subjects). The largest sample size in the simulation scenarios only had 20 total subjects (10 per group, 2 groups).

In order to assess the effect of sample size in our real dataset, we sampled 10 subjects from both the SS and CS groups and reran the analysis on this reduced dataset. The non-convergence rates for NBMM-PL and LMM increased to around 1% for both methods (Table 4). Surprisingly, the non-convergence rate for the NBMM-LP models changed very little even after reducing the number of subjects.

**Table 4:** Non-convergence rate, analysis run time, and number of DEGs for 4 hypothesis tests in the reduced shock dataset in which ten subjects from each group were randomly selected. The run time for fitting the full model for each gene, as well as the total time to fit models and perform hypothesis testing is displayed. There were 14,340 genes in the dataset and genes were labelled as a DEG if the Benjamini Hochberg adjusted *p*-value was < 0.05. For NBMM-LP, the percentage of genes in which one or more reduced models failed to converge is shown in parentheses after the full model non-convergence rate

	DESeq2	DESeq2*	edgeR	edgeR*	GEE	limma	LMM	NBMM-AGQ	NBMM-LP	NBMM-PL	rmRNAseq
% Non-converged	0%	0%	0%	0%	0.01%	0%	1.12%	0.03%	4.35% (9.07%)	1.28%	0%
**Analysis Time (minutes)**											
Model Fit Time	0.88	2.16	0.27	1.23	8.91	1.47	8.62	20.54	226.53	70.43	-
Total Time (Model Fit & Hypothesis Testing)	4.62	9.71	0.32	1.41	15.74	1.51	11.51	80.02	1067.07	73.26	327.84
**Between Subject Test**											
SS vs. CS (Any timepoint)	4,201	-	3,337	-	3,108	1,743	1,387	2,422	2,290	1,696	1,355
**Within Subject Tests**											
Change over time - SS group	1,684	4,466	1,397	3,960	4,875	5,894	3,925	5,124	4,390	4,323	2,994
Change over time - CS group	115	756	30	592	767	187	237	1,291	732	480	0
**Interaction Test**											
Any Significant Interaction Effect	166	719	117	701	773	740	398	1,291	748	549	129

#### Number of DEGs

Table 3 shows the number of DEGs identified by each method for various hypothesis tests using a 0.05 significance threshold for Benjamini Hochberg adjusted *p*-values. Though there was a range in the number of DEGs found across the different methods and tests, every method found the most DEGs for the test for the difference across time in the SS group. This is perhaps due in part to the fact that the SS group has more subjects than the CS group (*N*=21 vs. *N*=11). However, in the analysis of the reduced dataset in which each group was filtered to ten random subjects, this test still had the most DEGs across methods, while the test for differences across time in the CS group had the least amount of DEGs. This may indicate that the changes in gene expression over the course of treatment are more prevalent in SS patients than CS patients.

The differences in the number of DEGs for each method was generally what would be expected based on the results of the simulation study. NBMM-AGQ showed relatively inflated FDR values in the simulation study, and in this analysis this method found more DEGs than most other methods, particularly for the within-subject and interaction tests. DESeq2 and edgeR (ignoring correlation) had high DEG counts for the between-subject test and low DEG counts for the within-subject and interaction tests, which is also in line with the simulation results. limma also showed a mix of conservative and anti-conservative behavior in terms of the number of DEGs for each test. Finally, DESeq2*,edgeR*, NBMM-PL, NBMM-LP and LMM all had relatively moderate numbers of DEGs across all tests, with DESeq2*, edgeR*, NBMM-LP and NBMM-PL generally finding slightly more DEGs than LMM. This also corresponds to the simulation results in which in the largest sample size scenario (*N*=10 per group) all three methods exhibited FDR values close to the nominal rate with LMM showing conservative rates compared to the other three methods.

There were some discrepancies between this analysis and the simulation study. These discrepancies appear to be partially due to the difference in the number of subjects in the real data and the simulations and may point to the continuation of patterns related to sample size that were observed in the simulation study. For example, rmRNAseq displayed conservative FDR values and low power in the simulation study, though the power for the method increased with increasing numbers of subjects. In this analysis, the number of DEGs for rmRNAseq was comparable to other, less conservative methods, particularly for the between-subject test and the within-subject test for differences across time in the SS group. However, in the analysis of the reduced dataset, rmRNAseq found less DEGs than the majority of other methods (Table 4). Similarly, GEE generally had the most inflated FDR and highest power in the simulation study with FDR decreasing as the number of subjects increased. In this analysis the number of DEGs was moderate compared to the other methods, while in the analysis on the reduced data, GEE had more DEGs than most other methods, though NBMM-AGQ still found more DEGs for all tests except the between-subject test.

#### Hierarchical clustering and functional enrichment analysis results

**Fig. 3:**
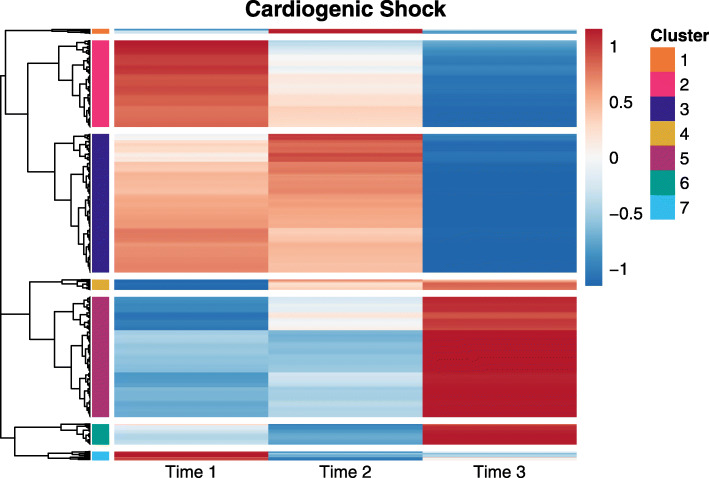
Heatmap of predicted gene expression (row scaled) across the three study timepoints for genes that were significant in a test for differential expression between any two timepoints in the CS group. Predicted values and significance results came from the LMM analysis. Genes are clustered using a correlation distance metric and complete linkage clustering methods and are split into seven clusters indicated by the color bars along the rows

For brevity, we will focus on results from our post-hoc analysis of genes with significant differential expression between at least two timepoints in the CS group. Similar results for the SS group can be found in Supple-mentary Fig. 5 and Supplementary Table 5. Using the LMM method, there were 1,003 genes that were significant for the test for differential expression between any two timepoints in the CS group. Figure 3 shows a heatmap of predicted expression (row scaled) for these genes along with the hierarchical clustering. Based on a visual inspection of the heatmap, a cutpoint was chosen such that the genes were split into seven clusters representing seven different patterns of change over time. For example, cluster 3 was the largest cluster with 328 genes. The expression of genes in this cluster stayed somewhat steady across the first two timepoints, but then steeply dropped between the second and third timepoint. Cluster 5 (309 genes) and cluster 2 (228 genes) were also relatively large. The genes in cluster 5 had expression levels that remained relatively unchanged between the first two timepoints, but then steeply climbed between the final two timepoints; cluster 2 contained genes that dropped in expression somewhat linearly across the three timepoints.

For three clusters (cluster 3, cluster 5, and cluster 6) at least one GO term was significantly enriched. Table 5 shows an abbreviated list of the significant terms. For cluster 3, several significantly enriched terms were related to an innate immune response including terms related to inflammation as well as neutrophil migration. For cluster 5, the GO terms were related to complement activation and phagocytosis. There were also terms related to adaptive immunity such as immunoglobulin production and positive regulation B-cell activation. Because genes from cluster 3 are relatively highly expressed at timepoints 1 and 2, but have lower expression at time 3, while cluster 5 shows the opposite behavior, these results may point to a heightened innate immune system response early in the ICU stay of CS patients, with a delayed adaptive immune response. Similar to cluster 5, genes in cluster 6 were involved in complement activation and phagocytosis. This cluster has a similar pattern across time to that of cluster 5, but genes in this category drop in expression between timepoints 1 and 2 before showing heightened expression at time 3.

**Table 5:** Functional enrichment analysis results. The 25 GO terms with the smallest Benjamini Hochberg (BH) adjusted *p*-values were selected for each cluster. The lists were then reduced to include only the most specific subclass for each ontology. All GO terms had a BH adjusted *p*-value < 0.01

GO Term	Description	# Genes in Set	# Genes in Cluster	# Expected	Fold Enrichment
**Cluster 3**					
GO:0002523	leukocyte migration involved in inflammatory response	12	6	0.30	20.00
GO:0050729	positive regulation of inflammatory response	86	13	2.15	6.05
GO:0051092	positive regulation of NF-kappaB transcription factor activity	136	16	3.40	4.71
GO:1990266	neutrophil migration	79	12	1.98	6.06
GO:0002755	MyD88-dependent toll-like receptor signaling pathway	33	7	0.83	8.43
GO:0045623	negative regulation of T-helper cell differentiation	14	5	0.35	14.29
GO:0060142	regulation of syncytium formation by plasma membrane fusion	14	5	0.35	14.29
GO:0071260	cellular response to mechanical stimulus	61	9	1.53	5.88
GO:0060396	growth hormone receptor signaling pathway	16	5	0.40	12.50
GO:0032651	regulation of interleukin-1 beta production	68	9	1.70	5.29
GO:0032695	negative regulation of interleukin-12 production	17	5	0.43	11.63
GO:0071354	cellular response to interleukin-6	28	6	0.70	8.57
**Cluster 5**					
GO:0006958	complement activation, classical pathway	83	40	2.11	18.96
GO:0030449	regulation of complement activation	71	29	1.80	16.11
GO:0002377	immunoglobulin production	128	34	3.25	10.46
GO:0038096	Fc-gamma receptor signaling pathway involved in phagocytosis	117	29	2.97	9.76
GO:0006910	phagocytosis, recognition	54	21	1.37	15.33
GO:0050871	positive regulation of B cell activation	111	25	2.82	8.87
**Cluster 6**					
GO:0006910	phagocytosis, recognition	54	9	0.22	40.91
GO:0006958	complement activation, classical pathway	83	9	0.34	26.47
GO:0006911	phagocytosis, engulfment	87	9	0.35	25.71
GO:0016584	nucleosome positioning	11	4	0.04	100.00
GO:0030261	chromosome condensation	35	5	0.14	35.71
GO:0045910	negative regulation of DNA recombination	37	4	0.15	26.67

## Discussion

In RNA-seq studies with longitudinal and other correlated designs, researchers are often interested in multiple hypotheses. Multiple DF tests allow researchers to assess multiple hypotheses at once, which is a useful method for selecting lists of genes for further exploration and can also be valuable in FDR control. Recently, several researchers have developed and compared analysis methods for analyzing longitudinal RNA-seq data. However, there has been little research evaluating and comparing these methods in the context of multiple DF testing. Understanding the comparative performance of various multiple DF hypothesis testing methods is becoming increasingly important as complex study designs become more common in correlated RNA-seq designs.

Of the methods compared in this study, LMM using data transformed using VST generally exhibited FDR closest to the nominal rate across the different sample sizes and multiple DF tests. NBMM-PL generally resulted in FDR values close to nominal as well, though slightly more inflated than LMM. GEE, NBMM-AGQ, and NBMM-LP had high FDR values across all simulation scenarios. DESeq2* and edgeR* had inflated FDR values at small sample sizes, but were relatively close to the nominal value for the highest sample size (*N*=10 per group). Conversely, limma had optimal FDR values at the smallest sample size, but these increased for the larger sample sizes. DESeq2 and edgeR (ignoring correlation) showed a mix of conservative and anti-conservative behavior. rmRNAseq had conservative FDR values, but was also extremely underpowered, particularly at the lower sample sizes. LMM and NBMM-PL generally had the highest power of the methods that had FDR values which were conservative or close to the nominal rate across all sample sizes and conditions.

Unsurprisingly, for the majority of methods, FDR values approached nominal rates and power increased as the sample size increased. We chose to use three small sample size scenarios in our simulation study because researchers often do not have the resources for large-scale studies, particularly in longitudinal studies where multiple samples are collected for each subject. However, we also analyzed data from a study involving shock patients and this study had 11 and 21 subjects in its two groups. In this analysis, methods such as GEE showed similar numbers of DEGs as LMM. When we reduced the dataset to 10 subjects per group, the difference in the number of DEGs for LMM compared to methods like GEE was wider. This implies that the FDR for methods that performed poorly, particularly at low sample sizes, may converge to that of LMM as the sample size increases past *N*=10 per group.

Another problem that occurred at low sample sizes was model non-convergence for LMM, NBMM-LP and NBMM-PL. Though LMM had the lowest non-convergence rate of these three methods, around 15% of models did not converge for this method at *N*=3 per group. We identified low between-subject variance, high dispersion, and small gene expression values as potential causes of non-convergence, though these data characteristics were not universal in non-converged models. Because LMM had otherwise good performance, future research regarding the cause of the high non-convergence rates and alternative ways of fitting singular and other non-converged models would be valuable. In small sample size cases in which many models do not converge, limma may be a good alternative because it demonstrated near nominal FDR at small sample sizes. However, no method was highly powered at the smallest sample size; choosing a sample size of at least 5 subjects per group is preferable.

One limitation of this study is that we only simulated data from one relatively simple correlation structure. This choice may have particularly affected the rmRNAseq simulation results since rmRNAseq utilizes a continuous autoregressive correlation structure and we simulated using a single random effect (equivalent to a compound symmetric structure). In analysis of the shock dataset, which may have a correlation structure that is not strictly compound symmetric, rmRNAseq did behave more similarly to other methods than in the simulation study, though we found that this was driven partially by sample size. Still, because complex RNA-seq studies are becoming more common, future research concerning the performance of multiple DF tests on data with different correlation structures and models with more complex random effects structures would be beneficial.

We did not explore the use of multiple DF tests in the context of single cell RNA-sequencing (scRNA-seq). Because gene expression of cells from the same sample or subject is more similar than cells from different samples [42], multi-sample scRNA-seq studies result in a hierarchical or correlated data structure, similar to longitudinal bulk RNA-seq studies. While the methods described in this work could theoretically be applied to scRNA-seq data, there are unique features of scRNA-seq data that could influence method performance and that should be further investigated. For example, scRNA-seq experiments typically collect data on thousands of cells from a relatively small number of samples or subjects, resulting in a large number of repeated observations per sample. This is in contrast to a longitudinal bulk RNA-seq study, where a relatively smaller number of repeated measurements (as few as two) is collected per subject. The library size per cell is also much smaller in scRNA-seq resulting in smaller numbers of counts per gene and more genes with zero counts. The data volume and sparsity could affect both the computation time and performance of the multiple DF testing methods. This would be a valuable area for future research.

## Conclusion

As the cost of RNA-seq experiments decreases, it becomes increasingly feasible to perform experiments using correlated designs, including longitudinal studies. Because these studies often involve multiple hypotheses and also require initial filtration to a set of genes for further exploration, multiple DF tests are a valuable tool for correlated RNA-seq data. In this work, we tested several modelling methods for longitudinal RNA-seq data with an emphasis on multiple DF hypotheses tests. Through a simulation study, we found that overall, LMM exhibited the best performance in terms of controlling FDR at nominal levels while maintaining the power to detect differential expression, though there were convergence issues at low sample sizes. limma offers a good alternative for small studies since it did not have convergence issues and had adequate FDR control at the smallest sample size. However, all methods were underpowered at *N*=3 per group, so we suggest that at least five subjects be included per group when possible.

Multiple DF testing is a valuable tool for selecting interesting genes for downstream analysis while also controlling the FDR. However, as we show in this study, there are many methods that allow for multiple DF testing all with different levels of efficacy. Making an informed decision when choosing a method based on the study goals as well as design elements such as sample size is key in producing useful, meaningful findings.

## Supplementary Information

**Additional file 1** Supplementary methods, results, tables, and figures.

## Data Availability

Code for simulating the datasets and running the methods used in the paper are available at https://github.com/ewynn610/multiDF_corr_RNASeqand through the corrRNASeq package, which can be found at https://github.com/ewynn610/corrRNASeq. Additional simulated datasets used in the simulation studies are available from the corresponding author upon request. The real RNA-Seq data was originally published in [39], and was downloaded for this application from the GEO DataSets website (GEO Dataset: GSE131411).

## Abbreviations

CPM: Counts Per Million
CS: Cardiogenic Shock
DF: Degrees of Freedom
FDR: False Discovery Rate
GEE: Generalized Estimating Equations
GLM: Generalized Linear Model
GLMM: Generalized Linear Mixed Model
GO: Gene Ontology
HPC: High Performance Computing
ICU: Intensive Care Unit
LMM: Linear Mixed Model
LRT: Likelihood Ratio Test
NBMM: Negative Binomial Mixed Model
NBMM-AGQ: Negative Binomial Mixed Model, Advanced Gaussian Quadrature approach
NBMM-LP: Negative Binomial Mixed Model, Laplace approach
NBMM-PL: Negative Binomial Mixed Model, Pseudolikelihood approach
RNA-seq: RNA-sequencing
SS: Septic Shock
TMM: Trimmed Mean of M-values
T1E: Type One Error
VST: Variance Stabilizing Transformation
