# Supplementary material for: A comparison of methods for multiple degree of freedom testing in repeated measures RNA-sequencing experiments
Source: BMC Med Res Methodol. 2022 May 28;22:153. doi: 10.1186/s12874-022-01615-8 (PMC9148455; doi:10.1186/s12874-022-01615-8)
Supplement: Supplementary file 1 — Additional file 1 Supplementary methods, results, tables, and figures. [file 12874_2022_1615_MOESM1_ESM.pdf]

A comparison of methods for multiple degree of freedom testing in repeated measures RNA-sequencing experiments
Supplementary Materials

1 Additional Information on Analysis Methods

1.1 limma

The `duplicateCorrelation` function in the `limma` package accounts for correlation between samples by estimating a common correlation for related samples across all genes and then incorporating this into the limma linear models[1]. In the context of a longitudinal or correlated RNA-Seq experiment, a single value is computed to represent the correlation between samples from the same subject or within the same cluster. This common value is then integrated into the linear model for every gene.

The moderated F-tests used in the limma pipeline use empirical Bayes methodology in which the genewise variance is shrunk towards a common variance and the new variance estimates are used to compute F statistics[2,3]. Degrees of freedom are also increased for the statistical tests to account for the additional information from other genes that is used via the empirical Bayes process. The moderated tests have decreased the false positive rate in genes with small variances, and increased the power to detect genes with high variance in comparison to traditional F-tests.

1.2 edgeR

The `edgeR` package employs a negative binomial generalized linear model (GLM) framework to address overdispersion[4,5]. Let Y_{gi} be the expression level of gene g in the ith sample, with $E(Y_{gi}) = \mu_{gi}$. In order to account for differences in library size, an offset, N_i, is calculated for each sample using the trimmed mean of M-values (TMM) method[4]. Then, the data can be modeled using

$$Y_{gi} \sim \mathcal{NB}(\mu_{gi}, \alpha_g) \quad (1)$$

$$log(\mu_{gi}) = \mathbf{X}_i\boldsymbol{\beta}_g + log(N_i) \quad (2)$$

where $\boldsymbol{\beta_g}$ is a vector of fixed effect regression coefficients for gene g, and the $\mathbf{X}_i$ are the fixed effects covariates for subject i. The genewise dispersion parameter α_g is estimated using an empirical Bayes approach[5].

1.3 DESeq2

The methodology behind the `DESeq2` package is very similar to that of `edgeR`, using the same model outlined in Equations 1-2 with the exception of the term N_i which is replaced with a size factor, s_i, which is calculated using the median ratio method[6,7]. The size factor is computed using

$$\hat{s}_i = \underset{g}{median} \frac{y_{gi}}{\left(\prod_{v=1}^{n} y_{gv}\right)^{1/(n)}} \quad (3)$$

where y_{gi} is the read counts for the gene g, subject i, and n is the total number of subjects. The fraction in this expression is the ratio of a single observation from gene g to the geometric mean across all samples for that gene. Then, for any subject i, the size factor is the median ratio across all genes. In the case that $y_{gi} = 0$, the corresponding gene is not used in the calculation of the median. Furthermore, zero valued

counts are excluded from the product in the denominator to avoid division by zero. For each raw count, y_{gi}, a corresponding scaled value, r_{gi} is then computed by dividing the raw count by its respective size factor such that $r_{gi} = \frac{y_{gi}}{\hat{s}^i}$.

The dispersion parameter α_g is estimated using a similar shrinkage estimation technique as `edgeR` which is outlined by Love, Huber, & Anders[7].

1.4 Generalized Estimating Equations (GEE)

Suppose Y_{gij} is the expression level of gene g in participant i at observation j. Then, like in GLM, in GEE modeling the relationship between the marginal mean, $E(Y_{gij}) = \mu_{gij}$ and a row vector of covariates $\mathbf{X_{ij}}$ can be represented using a link function h such that:

$$h(\mu_{gij}) = \mathbf{X}_{ij}\boldsymbol{\beta}_g \tag{4}$$

where $\boldsymbol{\beta}_g$ is, as before, a column vector of unknown regression coefficients. The variance in this model, $Var(Y_{gij})$, does not have to be fully specified, but instead is of the form $Var(Y_{gij}) = v(\mu_{gij})\phi_g$, where v is a known function and ϕ_g is a scale parameter, often unknown[8].

In order to account for the correlation between observations, a structure detailing the association between measurements within a subject must be specified. This structure, $\mathbf{R_{gi}}$, is called a working correlation structure and can take the same form as covariance structures used in other types of longitudinal modeling such as an exchangeable structure (covariance parameter is assumed to be the same between all observations within a subject), or an independent structure (covariance parameters between each pair of observations are estimated independently). For a subject i with m observations, if we let $\mathbf{A_{gi}} = diag(v(\mu_{gi1}), ..., v(\mu_{gim}))$, then the covariance matrix for $\mathbf{Y_{gi}}$ is specified by

$$\mathbf{V}_{gi} = \phi_g \mathbf{A}_{gi}^{1/2}\mathbf{R_{gi}}\mathbf{A}_{gi}^{1/2}. \tag{5}$$

Estimates for the regression coefficients $\hat{\boldsymbol{\beta}}_g$ can be obtained using $\boldsymbol{\mu}_g$ and $\mathbf{V}_g$. Let $\mathbf{D}_{gi} = \partial\boldsymbol{\mu}_{gi}/\partial\boldsymbol{\beta}_g^t$. Then estimates for the regression coefficients, $\hat{\boldsymbol{\beta}}_g$ can be found by solving the following equation (known as the generalized estimating equation):

$$\sum_{i=1}^{n} \mathbf{D_{gi}}^T \mathbf{V_{gi}}^{-1}(\mathbf{Y}_{gi} - \boldsymbol{\mu}_{gi}) = 0 \tag{6}$$

where n represents the number of subjects or clusters.

The covariance matrix for $\hat{\boldsymbol{\beta}}_g$ is typically estimated using robust (sandwich) estimators so that the estimates are robust to misspecifications of the working correlation matrix. If we estimate the covariance matrix of $\mathbf{Y}_{gi}$ using

$$Cov(\mathbf{Y}_{gi}) = (\mathbf{Y}_{gi} - \hat{\boldsymbol{\mu}}_{gi})(\mathbf{Y}_{gi} - \hat{\boldsymbol{\mu}}_{gi})^T \tag{7}$$

then the sandwich estimates for the covariance matrix for $\hat{\boldsymbol{\beta}}_g$ are found using

$$\mathbf{V}_g(\boldsymbol{\beta}_g) = \left(\sum_{i=1}^{n} \mathbf{D_{gi}}^T \mathbf{V_{gi}}^{-1}\mathbf{D_{gi}}\right)^{-1} \mathbf{M}_g \left(\sum_{i=1}^{n} \mathbf{D_{gi}}^T \mathbf{V_{gi}}^{-1}\mathbf{D_{gi}}\right)^{-1} \tag{8}$$

where

$$\mathbf{M}_g = \left(\sum_{i=1}^{n} \mathbf{D_{gi}}^T \mathbf{V_{gi}}^{-1} Cov(\mathbf{Y}_{gi}) \mathbf{V_{gi}}^{-1}\mathbf{D_{gi}}\right). \tag{9}$$

For this study we use a Poisson distribution to specify the mean of the data with the variance multiplied by a scale parameter to account for overdispersion. Thus, then $E(Y_{gij}) = \mu_{gij}$ and $Var(Y_{gij}) = \phi_g\mu_{gij}$. Often,

it is assumed that the correlation between each pair of observations is the same and so an exchangeable working correlation structure is used such that:

$$\mathbf{R}_{gi} = \begin{bmatrix} 1 & \rho_{gi} & \dots & \rho_{gi} \\ \rho_{gi} & 1 & \dots & \rho_{gi} \\ \vdots & \vdots & \ddots & \vdots \\ \rho_{gi} & \rho_{gi} & \dots & 1 \end{bmatrix} \tag{10}$$

1.4.1 Wang and Long's Small Sample Estimators

When applying GEE, the estimated covariance matrix for $\mathbf{Y}_{gi}$ is typically based only on data from the *ith* subject. Liang and Zeger[9] noted that given a common correlation structure across all subjects, then an estimated common correlation matrix $\mathbf{R_{gc}}$ can be found using

$$\mathbf{R_{gc}} = \frac{1}{\phi_g n} \sum_{i=1}^{n} \mathbf{A}_{gi}^{-1/2} (\mathbf{Y}_{gi} - \boldsymbol{\mu}_{gi})(\mathbf{Y_{gi}} - \boldsymbol{\mu}_{gi})^T \mathbf{A}_{gi}^{-1/2}. \tag{11}$$

Pan[10] suggested that if we can assume that there is common correlation structure across all subjects and that the variance for $\mathbf{Y_{gi}}$ is correctly specified, then an alternate estimate for the covariance matrix of $\mathbf{Y}_{gi}$ is given by

$$Cov_P(\mathbf{Y_{gi}}) = \phi \mathbf{A}_{gi}^{1/2} \mathbf{R}_{gc} \mathbf{A}_{gi}^{1/2} \tag{12}$$

$$= \mathbf{A}_{gi}^{1/2} \left(\frac{1}{n} \sum_{i=1}^{n} \mathbf{A}_{gi}^{-1/2} (\mathbf{Y_{gi}} - \boldsymbol{\mu}_{gi})(\mathbf{Y_{gi}} - \boldsymbol{\mu}_{gi})^T \mathbf{A}_{gi}^{-1/2} \right) \mathbf{A}_{gi}^{1/2} \tag{13}$$

This estimate is more efficient than the original covariance matrix estimator because it uses information from all subjects instead of just one. Therefore, when $Cov_P(\mathbf{Y_{gi}})$ is used in place of $Cov(\mathbf{Y_{gi}})$ when estimating the sandwich estimators, the resulting estimators for the covariance matrices of $\boldsymbol{\beta}_g$ are also more efficient. This is particularly true for small sample sizes.

In proposing a small sample estimator for the covariance matrix of $\hat{\boldsymbol{\beta}}_g$, Mancl and DeRouen[11] presented a method for correcting the bias in the covariance estimator for $\mathbf{Y_{gi}}$. The covariance estimator in Equation 7 is based on the residuals $\mathbf{r}_{gi} = \mathbf{Y}_{gi} - \hat{\boldsymbol{\mu}}_{gi}$. However, $\hat{\boldsymbol{\mu}}_{gi}$ is generally closer to $\mathbf{Y}_{gi}$ than $\boldsymbol{\mu}_{gi}$, resulting in biased residuals. Thus, using the originally proposed estimator for $Cov(\mathbf{Y}_{gi})$ when estimating the covariance matrix of $\hat{\boldsymbol{\beta}}_g$ results in estimates that are too small. This problem becomes exaggerated in small sample sizes.

To adjust for this bias, first note that the expected value of $Cov(\mathbf{Y_{gi}}) = \mathbf{r_{gi}} \mathbf{r}_{gi}^T$ can be approximated using

$$E(\mathbf{r_{gi}} \mathbf{r}_{gi}^T) = (\mathbf{I}_i - \mathbf{H}_{gii}) Cov(\mathbf{Y_{gi}}) (\mathbf{I}_i - \mathbf{H}_{gii})^T \tag{14}$$

where $\mathbf{I}_i$ is the $m \times m$ identity matrix for subject i with m observations, and

$$\mathbf{H}_{gii} = \mathbf{D_{gi}} \left(\sum_{i=1}^{n} \mathbf{D}_{gi}^T \mathbf{V}_{gi}^{-1} \mathbf{D}_{gi} \right)^{-1} \mathbf{D}_{gi}^T \mathbf{V}_{gi}^{-1}. \tag{15}$$

It follows that the covariance matrix for $\mathbf{Y}_i$ can be estimated using

$$Cov_M(\mathbf{Y}_{gi}) = (\mathbf{I}_i - \mathbf{H}_{gii}) \mathbf{r_{gi}} \mathbf{r}_{gi}^T (\mathbf{I}_i - \mathbf{H}_{gii})^T. \tag{16}$$

When $Cov_M(\mathbf{Y_{gi}})$ is used in place of $Cov(\mathbf{Y_{gi}})$ in Equation 9, the resulting covariance estimates for $\hat{\boldsymbol{\beta}}_g$ are less biased, especially in small sample sizes.

Wang and Long[8] sought to draw from the work of both Pan[10] and Mancl and DeRouen[11] by creating an estimator for the covariance of $\mathbf{Y}_{gi}$ that both corrected for bias and polled information from all subjects/clusters.

In Equation 13 the covariance of $\mathbf{Y}_{gi}$ is estimated using information from all subjects/clusters, but, the bias of the residuals is not accounted for. This can be accomplished by replacing $(\mathbf{Y_{gi}} - \boldsymbol{\mu})(\mathbf{Y_{gi}} - \boldsymbol{\mu})^T$ with Mancl's biased corrected estimator in Equation 16. Thus, the covariance estimator would be

$$Cov_W(\mathbf{Y}_{gi}) = \mathbf{A}_{gi}^{1/2} \frac{1}{n} \sum_{i=1}^{n} \mathbf{A}_{gi}^{-1/2} (\mathbf{I}_i - \mathbf{H}_{gii}) \mathbf{r_{gi}} \mathbf{r}_{gi}^T (\mathbf{I}_i - \mathbf{H}_{gii})^T \mathbf{A}_{gi}^{-1/2} \mathbf{A}_{gi}^{1/2} \tag{17}$$

where $\mathbf{r}_{gi} = \mathbf{Y}_{gi} - \hat{\boldsymbol{\mu}}_{gi}$. Like with the previous estimators, $Cov_W(\mathbf{Y_{gi}})$ is used in place of $Cov(\mathbf{Y_{gi}})$ in Equation 9 to generate a modified estimator for the covariance of $\hat{\boldsymbol{\beta}}_g$.

1.5 Negative Binomial Mixed Models (NBMM)

GLMM is an extension of generalized linear modeling (GLM) which allows the outcome variable to have a distribution other than the normal distribution, while also allowing for the inclusion of random effects. A link function h, such as a log function, is applied to the expected value of the outcome in order to make it continuous, and $h(\mu)$, where μ is the expected value of the outcome, can be modeled linearly with fixed and random effects.

Because RNA-Seq data are over-dispersed counts, they are often modeled using a negative binomial distribution. Thus, in using a GLMM to model correlated RNA-Seq data, a negative binomial mixed model (NBMM) can be used. If we assume, as previously, that we have n subjects and m observations per subject and that Y_{gij} is the expression value for gene g from subject i at observation j, then using a log link function, we can write

$$\begin{aligned} Y_{gij} &\sim \mathcal{NB}(\mu_{gij}, \alpha_g) && (18)\\ log(\mu_{gij}) &= \mathbf{X}_{ij}\boldsymbol{\beta_g} + \mathbf{Z}_{ij}\mathbf{b}_{gi} && (19)\\ b_{gi} &\sim \mathcal{MVN}(\mathbf{0}, \boldsymbol{\Sigma}_{gb}) && (20) \end{aligned}$$

where α_g is the dispersion parameter such that $Var(Y_{gij}) = \mu_{gij} + \alpha_g \mu_{gij}^2$. All other notation is the same as outlined for equations 28-30.

1.5.1 Maximum Likelihood Approach: Laplace Approximation

Because NBMM's are not linear, the likelihood function often involves high dimensional integrals to which there are no closed form solutions[12]. Thus, in using a maximum likelihood approach, we must use approximation techniques. One such technique is automatic differentiation (AD), which utilizes a Laplace approximation to estimate model parameters. Fournier et al.[13] constructed an AD model builder (ADMB), a series of tools built specifically for likelihood based parameter estimation and model building for non-linear models.

1.5.2 Maximum Likelihood Approach: Adaptive Gaussian Quadrature

Adaptive Gaussian Quadrature is an alternative to Laplace approximation which still uses a maximum likelihood estimation approach. This method is often more accurate, but also more computationally expensive than the Laplace method.[14]. More details on this approximation technique and its application to RNA-seq data can be found in the recent work of Tsonaka & Spitali[14].

1.5.3 Pseudo-Likelihood Approach

Estimating negative binomial mixed model (NBMM) parameters using a pseudo-likelihood approach (NBMM-PL) involves computing linearized "pseudo-data" and model weights and then iteratively alternating between fitting a weighted LMM using the pseudo-data and recomputing the pseudo-data and weights until model convergence.

The basis of this approach relies on the fact that the negative binomial model can be approximated by a weighted normal model[15]. Suppose Y_{gij} is the expression level of gene g in subject i at observation j with

$E(Y_{gij}) = \mu_{gij}$. An NBMM can be specified by:

$$Y_{gij} \sim \mathcal{NB}(\mu_{gij}, \alpha_g) \quad (21)$$
$$log(\mu_{gij}) = \mathbf{X}_{ij}\boldsymbol{\beta_g} + \mathbf{Z}_{ij}\mathbf{b}_{gi} + \rho_{ij} \quad (22)$$
$$b_{gi} \sim \mathcal{MVN}(\mathbf{0}, \mathbf{\Sigma}_{gb}) \quad (23)$$

where $\mathbf{X}_{ij}$ and $\mathbf{Z}_{ij}$ are row vectors of fixed random effects respectively, $\boldsymbol{\beta_g}$ is a column vector the gene specific fixed effect coefficients, and $\mathbf{b}_{gi}$ is a column vector of random effects for gene g and subject i. We assume that for each gene g, the subject-specific random effects have a multivariate normal distribution with mean $\mathbf{0}$ and variance-covariance matrix $\mathbf{\Sigma}_{bg}$. Finally, ρ_{ij} is an offset for subject i at observation j. Then

$$\mathcal{NB}(\mu_{gij}, \alpha_g) \approx \mathcal{N}(\eta_{gij}, w_{gij}^{-1}\sigma_g^2) \quad (24)$$

where $log(\mu_{gij}) = \eta_{gij} = \mathbf{X}_{ij}\boldsymbol{\beta}_g + \mathbf{Z}_{ij}\mathbf{b}_{gi}$ and w_{gij} is a weight for gene g, subject i, and observation j. If $\mathbf{P_g} \sim \mathcal{N}(\boldsymbol{\eta_g}, \mathbf{w_g}^{-1}\sigma_g^2)$ represents a set of "pseudo-data" for gene g, and $\mathbf{P_g}$ is modeled using a weighted LMM such that

$$p_{gij} = \mathbf{X}_{ij}\boldsymbol{\beta_g} + \mathbf{Z}_{ij}\mathbf{b}_{gi} + w_{gij}^{-1/2}\epsilon_{gij} \quad (25)$$
$$b_{gi} \sim \mathcal{MVN}(\mathbf{0}, \mathbf{\Sigma}_{gb}) \quad (26)$$
$$\epsilon_{gij} \sim \mathcal{N}(0, \sigma_g^2), \quad (27)$$

then this model can be used to approximate a NBMM for $\mathbf{Y_g} \sim \mathcal{NB}(\boldsymbol{\mu_g}, \alpha_g)$.

In the weighted LMM model, both the calculation of the pseudo-data ($\mathbf{P_g}$) and the weights ($\mathbf{w_g}$) depend on the parameters $\boldsymbol{\eta_g}$ and α_g, which are both unknown. Thus, in order to fit an approximate model using the pseudo-data and weights, the following iterative approach is used:

1. A negative binomial GLM is fit in order to obtain initial estimates for $\boldsymbol{\eta_g}$ and α_g.

2. Pseudo-data ($\mathbf{P_g}$) and the weights ($\mathbf{w_g}$) are calculated using the estimates for $\boldsymbol{\eta_g}$ and α_g.

3. A weighted LMM is fit using the current values of $\mathbf{P_g}$ and $\mathbf{w_g}$.

4. Using the results from the weighted LMM, a new estimate for $\boldsymbol{\eta_g}$ is computed as well as a new estimate for α_g, which is found using a Newton-Raphson algorithm.

5. Steps 2-4 are repeated until convergence.

1.6 Linear Mixed Models (LMM)

Assume we have RNA-Seq count data from n subjects and m timepoints. Let Y_{gij} be the expression value for gene g from subject i at time point or observation j, then

$$Y_{gij} \sim \mathcal{N}(\mu_{gij}, \sigma_g^2) \quad (28)$$
$$\mu_{gij} = \mathbf{X}_{ij}\boldsymbol{\beta_g} + \mathbf{Z}_{ij}\mathbf{b}_{gi} \quad (29)$$
$$\mathbf{b_{gi}} \sim \mathcal{MVN}(\mathbf{0}, \mathbf{\Sigma}_{gb}) \quad (30)$$

where μ_{gij} is the mean expression value for gene g in subject i at observation j and σ_g^2 is the variance for gene g. $\boldsymbol{\beta_g}$ is a column vector of fixed effect regression coefficients for gene g, $\mathbf{X}_{ij}$ is a row vector of fixed effects covariates for subject i at observation j, $\mathbf{Z}_{ij}$ is a row vector of random effects covariates for subject i at observation j, and $\mathbf{b}_{gi}$ is a column vector of random effects for gene g and subject i. We assume that for each gene g, the subject-specific random effects have a multivariate normal distribution with mean $\mathbf{0}$ and variance-covariance matrix $\mathbf{\Sigma}_{bg}$.

Of course, this model assumes a normally distributed, continuous outcome, but RNA-Seq data are over-dispersed counts. Furthermore, we see from Equation 28 that for any given gene g, the variance σ_g^2 is constant across all subjects and observations. However, there is typically a mean-variance relationship in RNA-Seq data, with larger mean expression values corresponding to larger variance. One approach to

mitigate these issues is applying a transformation to the RNA-Seq counts. One such transformation is the variance-stabilizing transformation in the `DESeq2` package[7]. This transformation both scales RNA-Seq counts and makes the variance approximately independent of the mean[6]. To scale the data to account for differences in sequencing depth between samples, a size factor, $\hat{s}_{ij}$, for subject i, timepoint j is computed using 3.

Using the scaled data, an additional transformation is performed to eliminate the mean-variance relationship in the data. If we let $v(\mu_{gij})$ be the mean variance relationship estimated by `DESeq2`, then the variance stabilizing transformation for r_{gij} is given by

$$r_{gij}^{vst} = \int_0^{r_{gij}} \frac{1}{\sqrt{v(\mu_{gij})}} d\mu_{gij}. \tag{31}$$

Given that there are not a large number of zero or low count genes, the VST transformation generally results in data that is roughly normal and with a variance that is approximately the same across all values[6,16]. However, if there are a large number of zero or low count genes, the variance may not stabilize even after performing VST and thus a linear model may not be appropriate for the transformed data[17].

Assuming that the model assumptions are met for the VST counts, we can use the LMM outlined in equations 28-30 to model the transformed data.

1.6.1 Satterthwaite Degrees of Freedom

The Satterthwaite method uses Satterthwaite's method of moments technique to approximate the DF[18,19]. This method can be used to approximate DF for t-tests and denominator DF for F-tests[20,21,22]. Suppose that we are testing the hypothesis $H_0 : \mathbf{L}\boldsymbol{\beta} = 0$, where $\mathbf{L}$ is a contrast matrix and $\boldsymbol{\beta}$ is a vector of fixed effect parameters for a LMM. If $Rank(\mathbf{L}) = 1$, then a t-test is appropriate, and otherwise an F-test can be used. In this context, the covariance matrix of $\boldsymbol{\beta}$ is

$$\mathbf{C}(\boldsymbol{\theta}) = (\mathbf{X}^T\mathbf{V}(\boldsymbol{\theta})\mathbf{X})^{-1} \tag{32}$$

where $\mathbf{X}$ is a vector of fixed effects covariates and $\mathbf{V}(\theta)$ is the variance of the outcome for the LMM with $\boldsymbol{\theta}$ representing the parameters for the fixed effect variance and residual error variance. This covariance matrix can be estimated by $\hat{\mathbf{C}} = \mathbf{C}(\hat{\boldsymbol{\theta}})$. If $Rank(\mathbf{L}) = 1$, the t-statistic for this hypothesis would be

$$t = \frac{\mathbf{L}\boldsymbol{\beta}}{\mathbf{L}\hat{\mathbf{C}}\mathbf{L}^T}. \tag{33}$$

If t is distributed according to a t-distribution with q DF, then, based on the relationship between the t-distribution and χ^2 distribution, the distribution of the statistic

$$Q = \frac{q\mathbf{L}\hat{\mathbf{C}}\mathbf{L}^T}{\mathbf{L}\mathbf{C}(\boldsymbol{\theta})\mathbf{L}^T} \tag{34}$$

can be approximated using a χ_q^2 distribution. Using Satterthwaite's method of moments technique, we can equate the variance of the test statistic, χ^2, to the theoretical variance of a χ_q^2 distribution and then solve for q. Thus

$$2q = Var\left(\frac{q\mathbf{L}\hat{\mathbf{C}}\mathbf{L}^T}{\mathbf{L}\mathbf{C}(\boldsymbol{\theta})\mathbf{L}^T}\right) \tag{35}$$

$$q = \frac{2\mathbf{L}\mathbf{C}(\boldsymbol{\theta})\mathbf{L}^T}{Var(\mathbf{L}\hat{\mathbf{C}}\mathbf{L})}. \tag{36}$$

The process for finding the DF when $Rank(\mathbf{L}) > 1$ is similar to the process for when $Rank(\mathbf{L}) = 1$. Suppose that $p = Rank(\mathbf{L})$. Then, the distribution of the statistic

$$F = \frac{1}{p}[\mathbf{L}\hat{\boldsymbol{\beta}}]^T(\mathbf{L}\hat{\mathbf{C}}\mathbf{L}^T)^{-1}\mathbf{L}\hat{\boldsymbol{\beta}} \tag{37}$$

can be approximated by an $F_{p,q}$ distribution, where p and q are the numerator and denominator DF respectively. Then using Satterthwaite's method of moments technique, the approximation for q can be found by setting the estimated expected value of F equal to the theoretical expectation for an $F_{p,q}$ distribution:

$$\frac{q}{q-2} = E(F) \tag{38}$$

$$m = \frac{2E(F)}{E(F)-1}. \tag{39}$$

We also use Satterthwaite DF approximation for the NBMM-PL models.

1.7 rmRNASeq

The method employed in the rmRNASeq package is similar to the LMM method with a few key differences, First, rmRNASeq uses a voom transformation rather than using a VST. Second, rmRNASeq uses a continous autoregreesive correlation structure where with LMM we use random effects. Finally, rmRNASeq utilizes a parametric bootstrapping procedure to calculate p-values. More information on the details of this method can be found in the paper introducing the package by Nguyen & Nettleton [23].

2 Additional Simulation Results

2.1 Non-Convergence Exploration

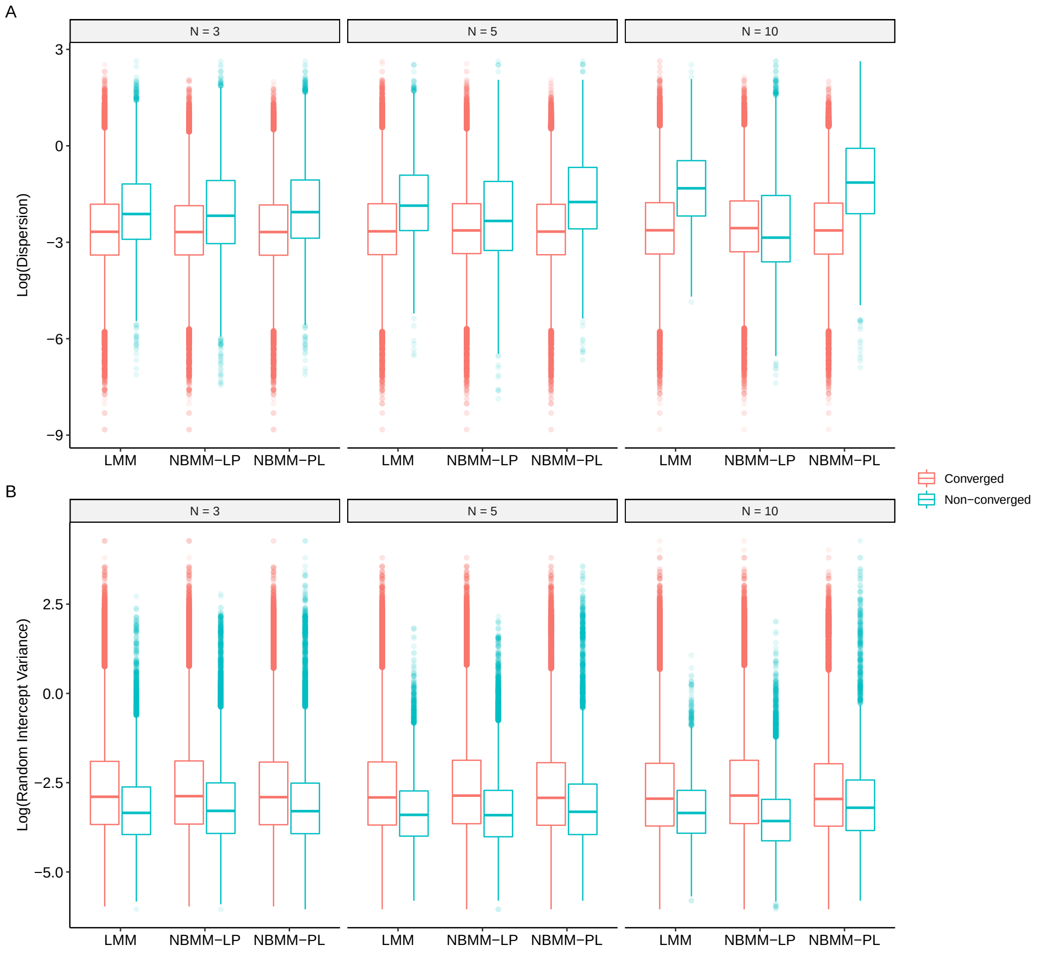

Figure 1: Boxplots of simulation parameters for converged vs. non-converged models across the three sample size scenarios. Methods in which less than 1% of models failed to converge are not included in the figure.

Table 1: Non-convergence rate, analysis run time, and number of DEGs for 4 hypothesis tests in the reduced shock dataset in which ten subjects from each group were randomly selected. The run time for fitting the full model for each gene, as well as the total time to fit models and perform hypothesis testing is displayed. There were 14,340 genes in the dataset and genes were labelled as a DEG if the Benjamini Hochberg adjusted p-value was < 0.05.

	n3		n5		n10	
	CPM Filter = 1	CPM Filter = 5	CPM Filter = 1	CPM Filter = 5	CPM Filter = 1	CPM Filter = 5
LMM	14.96%	14.07%	8.12%	7.33%	3.65%	3.08%
NBMM-LP	20.62%	19.89%	15.1%	15.06%	11.51%	12.32%
NBMM-PL	15.57%	14.5%	8.67%	7.65%	4.04%	3.29%

2.2 Type 1 Error Rate

Table 2: Type one error rate across several testing thresholds for each sample size and hypothesis test. Mean (Range) across the 10 simulated datasets.

	Difference Between Groups at Any Timepoint											
	n3				n5				n10			
	0.001	0.01	0.05	0.1	0.001	0.01	0.05	0.1	0.001	0.01	0.05	0.1
DESeq2	0.027 (0.025, 0.031)	0.059 (0.055, 0.064)	0.12 (0.115, 0.124)	0.172 (0.166, 0.178)	0.022 (0.021, 0.024)	0.054 (0.051, 0.056)	0.114 (0.11, 0.118)	0.165 (0.16, 0.169)	0.02 (0.017, 0.021)	0.049 (0.044, 0.05)	0.107 (0.105, 0.109)	0.159 (0.154, 0.165)
edgeR	0.02 (0.019, 0.022)	0.046 (0.043, 0.049)	0.096 (0.092, 0.098)	0.139 (0.136, 0.143)	0.019 (0.017, 0.02)	0.045 (0.042, 0.046)	0.097 (0.095, 0.1)	0.143 (0.139, 0.146)	0.02 (0.017, 0.021)	0.046 (0.041, 0.048)	0.1 (0.097, 0.102)	0.148 (0.144, 0.151)
GEE	0.44 (0.43, 0.456)	0.522 (0.512, 0.537)	0.606 (0.597, 0.621)	0.656 (0.65, 0.664)	0.082 (0.078, 0.084)	0.146 (0.141, 0.15)	0.246 (0.239, 0.25)	0.319 (0.31, 0.324)	0.015 (0.013, 0.017)	0.047 (0.044, 0.052)	0.119 (0.115, 0.127)	0.185 (0.179, 0.192)
limma	0.002 (0.001, 0.002)	0.013 (0.009, 0.016)	0.056 (0.042, 0.071)	0.106 (0.083, 0.127)	0.002 (0.002, 0.004)	0.016 (0.011, 0.023)	0.066 (0.05, 0.092)	0.121 (0.095, 0.157)	0.003 (0.002, 0.006)	0.018 (0.013, 0.034)	0.071 (0.051, 0.108)	0.13 (0.099, 0.183)
LMM	0 (0, 0)	0.003 (0.002, 0.004)	0.03 (0.027, 0.032)	0.075 (0.071, 0.079)	0 (0, 0.001)	0.007 (0.006, 0.008)	0.041 (0.038, 0.045)	0.091 (0.087, 0.095)	0.001 (0, 0.001)	0.008 (0.007, 0.01)	0.047 (0.045, 0.05)	0.096 (0.092, 0.1)
NBMM-AGQ	0.019 (0.017, 0.021)	0.06 (0.057, 0.064)	0.148 (0.142, 0.155)	0.222 (0.217, 0.228)	0.029 (0.026, 0.031)	0.056 (0.053, 0.062)	0.116 (0.111, 0.122)	0.175 (0.168, 0.182)	0.03 (0.029, 0.032)	0.042 (0.04, 0.044)	0.084 (0.082, 0.087)	0.133 (0.129, 0.135)
NBMM-LP	0.013 (0.011, 0.014)	0.059 (0.056, 0.062)	0.168 (0.165, 0.172)	0.259 (0.254, 0.262)	0.005 (0.005, 0.006)	0.03 (0.028, 0.031)	0.106 (0.103, 0.109)	0.181 (0.176, 0.185)	0.002 (0.002, 0.003)	0.019 (0.017, 0.02)	0.075 (0.072, 0.078)	0.134 (0.132, 0.136)
NBMM-PL	0 (0, 0.001)	0.005 (0.003, 0.006)	0.038 (0.034, 0.041)	0.09 (0.083, 0.094)	0.001 (0, 0.001)	0.01 (0.008, 0.011)	0.052 (0.049, 0.058)	0.109 (0.105, 0.113)	0.001 (0.001, 0.002)	0.012 (0.011, 0.015)	0.059 (0.057, 0.061)	0.114 (0.11, 0.117)
rmRNAseq	0 (0, 0.001)	0.003 (0.003, 0.004)	0.018 (0.016, 0.02)	0.039 (0.037, 0.041)	0 (0, 0)	0 (0, 0.001)	0.004 (0.003, 0.005)	0.011 (0.01, 0.013)	0 (0, 0)	0 (0, 0)	0.001 (0.001, 0.002)	0.005 (0.004, 0.006)

	Difference Between Any Timepoints in the Treatment Group											
	n3				n5				n10			
	0.001	0.01	0.05	0.1	0.001	0.01	0.05	0.1	0.001	0.01	0.05	0.1
DESeq2	0.001 (0.001, 0.002)	0.007 (0.006, 0.008)	0.027 (0.025, 0.03)	0.051 (0.047, 0.055)	0.001 (0, 0.001)	0.005 (0.004, 0.006)	0.022 (0.02, 0.025)	0.044 (0.039, 0.046)	0.001 (0, 0.001)	0.004 (0.003, 0.005)	0.019 (0.018, 0.022)	0.041 (0.038, 0.043)
DESeq2*	0.006 (0.005, 0.007)	0.024 (0.022, 0.025)	0.069 (0.066, 0.074)	0.117 (0.111, 0.126)	0.003 (0.003, 0.004)	0.017 (0.015, 0.02)	0.061 (0.058, 0.066)	0.109 (0.106, 0.113)	0.002 (0.001, 0.003)	0.013 (0.011, 0.015)	0.054 (0.051, 0.058)	0.104 (0.1, 0.111)
edgeR	0.001 (0.001, 0.002)	0.006 (0.005, 0.007)	0.021 (0.019, 0.022)	0.04 (0.038, 0.042)	0.001 (0, 0.001)	0.004 (0.003, 0.005)	0.018 (0.017, 0.02)	0.037 (0.033, 0.04)	0.001 (0, 0.001)	0.004 (0.003, 0.004)	0.017 (0.015, 0.019)	0.037 (0.035, 0.038)
edgeR*	0.005 (0.004, 0.006)	0.018 (0.016, 0.02)	0.055 (0.053, 0.06)	0.094 (0.089, 0.1)	0.003 (0.002, 0.003)	0.014 (0.012, 0.016)	0.05 (0.047, 0.055)	0.092 (0.089, 0.097)	0.002 (0.001, 0.002)	0.011 (0.009, 0.013)	0.048 (0.044, 0.051)	0.094 (0.09, 0.099)
GEE	0.24 (0.234, 0.246)	0.312 (0.306, 0.32)	0.399 (0.392, 0.407)	0.457 (0.451, 0.463)	0.07 (0.067, 0.073)	0.124 (0.12, 0.127)	0.208 (0.202, 0.212)	0.272 (0.265, 0.279)	0.026 (0.022, 0.028)	0.057 (0.053, 0.061)	0.124 (0.118, 0.131)	0.184 (0.177, 0.192)
limma	0.001 (0, 0.002)	0.011 (0.009, 0.015)	0.054 (0.042, 0.071)	0.103 (0.087, 0.133)	0.002 (0.001, 0.004)	0.017 (0.011, 0.028)	0.07 (0.049, 0.108)	0.128 (0.094, 0.183)	0.005 (0.002, 0.013)	0.028 (0.012, 0.06)	0.096 (0.059, 0.157)	0.164 (0.112, 0.245)
LMM	0.001 (0.001, 0.002)	0.011 (0.01, 0.012)	0.054 (0.052, 0.058)	0.107 (0.102, 0.115)	0.001 (0, 0.002)	0.011 (0.009, 0.014)	0.052 (0.05, 0.057)	0.103 (0.1, 0.108)	0.001 (0.001, 0.002)	0.01 (0.008, 0.012)	0.05 (0.046, 0.052)	0.101 (0.096, 0.106)
NBMM-AGQ	0.025 (0.023, 0.027)	0.072 (0.068, 0.079)	0.168 (0.162, 0.177)	0.249 (0.244, 0.257)	0.027 (0.023, 0.029)	0.053 (0.048, 0.056)	0.12 (0.114, 0.125)	0.187 (0.18, 0.193)	0.017 (0.016, 0.019)	0.031 (0.03, 0.033)	0.083 (0.079, 0.087)	0.141 (0.137, 0.148)
NBMM-LP	0.011 (0.011, 0.012)	0.054 (0.051, 0.055)	0.155 (0.15, 0.16)	0.242 (0.237, 0.245)	0.004 (0.003, 0.005)	0.027 (0.026, 0.029)	0.097 (0.095, 0.1)	0.17 (0.166, 0.175)	0.002 (0.002, 0.002)	0.016 (0.014, 0.017)	0.068 (0.065, 0.071)	0.129 (0.125, 0.132)
NBMM-PL	0.002 (0.001, 0.002)	0.015 (0.014, 0.017)	0.066 (0.062, 0.069)	0.127 (0.123, 0.135)	0.002 (0.001, 0.003)	0.016 (0.013, 0.019)	0.066 (0.064, 0.072)	0.124 (0.12, 0.128)	0.002 (0.002, 0.003)	0.015 (0.013, 0.017)	0.064 (0.059, 0.068)	0.12 (0.113, 0.126)
rmRNAseq	0 (0, 0)	0 (0, 0.001)	0.003 (0.002, 0.004)	0.011 (0.009, 0.012)	0 (0, 0)	0 (0, 0)	0.001 (0, 0.002)	0.005 (0.003, 0.006)	0 (0, 0)	0 (0, 0)	0.001 (0, 0.001)	0.003 (0.002, 0.004)

Table 2: Type one error rate across several testing thresholds for each sample size and hypothesis test. Mean (Range) across the 10 simulated datasets (continued).

	Any Significant Interaction Effect											
	n3				n5				n10			
	0.001	0.01	0.05	0.1	0.001	0.01	0.05	0.1	0.001	0.01	0.05	0.1
DESeq2	0.002 (0.001, 0.002)	0.008 (0.006, 0.009)	0.028 (0.025, 0.03)	0.053 (0.048, 0.055)	0.001 (0, 0.001)	0.005 (0.004, 0.006)	0.021 (0.02, 0.023)	0.043 (0.041, 0.046)	0 (0, 0.001)	0.004 (0.003, 0.004)	0.02 (0.018, 0.021)	0.042 (0.04, 0.045)
DESeq2*	0.007 (0.006, 0.008)	0.025 (0.024, 0.027)	0.072 (0.069, 0.074)	0.119 (0.115, 0.121)	0.004 (0.003, 0.005)	0.018 (0.015, 0.02)	0.061 (0.056, 0.065)	0.11 (0.106, 0.114)	0.002 (0.001, 0.002)	0.014 (0.012, 0.015)	0.055 (0.051, 0.058)	0.104 (0.101, 0.109)
edgeR	0.001 (0.001, 0.002)	0.006 (0.005, 0.006)	0.022 (0.02, 0.023)	0.041 (0.038, 0.043)	0.001 (0, 0.001)	0.004 (0.003, 0.005)	0.018 (0.017, 0.018)	0.036 (0.035, 0.039)	0 (0, 0.001)	0.004 (0.003, 0.004)	0.018 (0.017, 0.019)	0.038 (0.036, 0.041)
edgeR*	0.005 (0.005, 0.006)	0.02 (0.019, 0.02)	0.057 (0.055, 0.058)	0.096 (0.094, 0.099)	0.003 (0.002, 0.003)	0.014 (0.012, 0.016)	0.05 (0.046, 0.055)	0.092 (0.09, 0.097)	0.002 (0.001, 0.002)	0.011 (0.01, 0.013)	0.049 (0.047, 0.052)	0.094 (0.09, 0.098)
GEE	0.172 (0.166, 0.18)	0.242 (0.239, 0.254)	0.336 (0.333, 0.345)	0.401 (0.395, 0.409)	0.037 (0.035, 0.039)	0.081 (0.078, 0.083)	0.161 (0.157, 0.165)	0.226 (0.222, 0.23)	0.009 (0.008, 0.011)	0.033 (0.03, 0.036)	0.093 (0.088, 0.097)	0.153 (0.147, 0.157)
limma	0.001 (0.001, 0.002)	0.011 (0.008, 0.012)	0.05 (0.039, 0.056)	0.096 (0.079, 0.107)	0.001 (0.001, 0.003)	0.013 (0.008, 0.023)	0.059 (0.046, 0.091)	0.111 (0.09, 0.155)	0.002 (0.001, 0.004)	0.016 (0.012, 0.024)	0.068 (0.05, 0.087)	0.125 (0.098, 0.156)
LMM	0.001 (0.001, 0.002)	0.011 (0.01, 0.013)	0.054 (0.051, 0.057)	0.107 (0.104, 0.112)	0.001 (0.001, 0.002)	0.01 (0.009, 0.012)	0.052 (0.05, 0.056)	0.103 (0.101, 0.108)	0.001 (0.001, 0.002)	0.01 (0.009, 0.012)	0.05 (0.047, 0.053)	0.1 (0.096, 0.105)
NBMM-AGQ	0.026 (0.023, 0.03)	0.073 (0.07, 0.078)	0.172 (0.168, 0.177)	0.253 (0.247, 0.26)	0.026 (0.024, 0.03)	0.052 (0.047, 0.058)	0.12 (0.113, 0.125)	0.187 (0.18, 0.193)	0.017 (0.015, 0.018)	0.032 (0.029, 0.034)	0.083 (0.08, 0.086)	0.142 (0.139, 0.145)
NBMM-LP	0.012 (0.011, 0.013)	0.055 (0.053, 0.056)	0.158 (0.155, 0.16)	0.245 (0.242, 0.249)	0.005 (0.004, 0.005)	0.029 (0.028, 0.03)	0.1 (0.097, 0.105)	0.171 (0.167, 0.178)	0.002 (0.002, 0.003)	0.018 (0.017, 0.02)	0.071 (0.068, 0.074)	0.13 (0.129, 0.132)
NBMM-PL	0.002 (0.001, 0.003)	0.015 (0.014, 0.017)	0.068 (0.064, 0.07)	0.127 (0.122, 0.132)	0.002 (0.001, 0.003)	0.015 (0.013, 0.017)	0.066 (0.063, 0.071)	0.125 (0.122, 0.131)	0.002 (0.002, 0.003)	0.016 (0.014, 0.017)	0.064 (0.06, 0.067)	0.12 (0.116, 0.125)
rmRNAseq	0 (0, 0.001)	0.002 (0.002, 0.002)	0.012 (0.01, 0.013)	0.026 (0.025, 0.028)	0 (0, 0)	0 (0, 0)	0.003 (0.002, 0.003)	0.008 (0.007, 0.009)	0 (0, 0)	0 (0, 0)	0.001 (0, 0.002)	0.004 (0.003, 0.005)

	Any Significant Coefficient											
	n3				n5				n10			
	0.001	0.01	0.05	0.1	0.001	0.01	0.05	0.1	0.001	0.01	0.05	0.1
DESeq2	0.022 (0.019, 0.025)	0.048 (0.045, 0.05)	0.096 (0.09, 0.101)	0.139 (0.134, 0.144)	0.016 (0.015, 0.017)	0.039 (0.036, 0.041)	0.085 (0.083, 0.088)	0.127 (0.123, 0.13)	0.014 (0.011, 0.015)	0.034 (0.03, 0.036)	0.077 (0.074, 0.079)	0.117 (0.115, 0.121)
DESeq2*	0.012 (0.01, 0.013)	0.035 (0.032, 0.036)	0.086 (0.084, 0.088)	0.134 (0.128, 0.139)	0.005 (0.004, 0.006)	0.023 (0.021, 0.026)	0.07 (0.066, 0.076)	0.12 (0.117, 0.125)	0.003 (0.002, 0.004)	0.016 (0.014, 0.017)	0.059 (0.056, 0.063)	0.109 (0.103, 0.116)
edgeR	0.016 (0.015, 0.018)	0.037 (0.035, 0.039)	0.075 (0.071, 0.078)	0.108 (0.102, 0.112)	0.014 (0.012, 0.015)	0.032 (0.03, 0.034)	0.071 (0.069, 0.073)	0.107 (0.103, 0.109)	0.014 (0.011, 0.016)	0.032 (0.029, 0.035)	0.071 (0.067, 0.074)	0.108 (0.105, 0.111)
edgeR*	0.009 (0.008, 0.01)	0.026 (0.024, 0.028)	0.066 (0.064, 0.067)	0.105 (0.102, 0.107)	0.004 (0.003, 0.005)	0.018 (0.016, 0.02)	0.056 (0.051, 0.062)	0.098 (0.095, 0.104)	0.002 (0.002, 0.002)	0.013 (0.011, 0.015)	0.051 (0.047, 0.054)	0.096 (0.091, 0.099)
GEE	0.591 (0.584, 0.604)	0.664 (0.656, 0.676)	0.734 (0.724, 0.746)	0.771 (0.764, 0.78)	0.155 (0.151, 0.158)	0.24 (0.236, 0.247)	0.351 (0.346, 0.353)	0.424 (0.418, 0.428)	0.044 (0.039, 0.047)	0.091 (0.085, 0.096)	0.18 (0.172, 0.187)	0.251 (0.241, 0.259)
limma	0.001 (0.001, 0.002)	0.011 (0.009, 0.014)	0.052 (0.044, 0.059)	0.103 (0.085, 0.116)	0.002 (0.002, 0.003)	0.015 (0.012, 0.022)	0.065 (0.05, 0.083)	0.121 (0.094, 0.151)	0.003 (0.001, 0.008)	0.021 (0.013, 0.042)	0.08 (0.057, 0.134)	0.142 (0.107, 0.213)
LMM	0.001 (0, 0.001)	0.008 (0.007, 0.009)	0.048 (0.044, 0.051)	0.099 (0.095, 0.104)	0.001 (0, 0.001)	0.009 (0.008, 0.011)	0.048 (0.046, 0.052)	0.101 (0.096, 0.107)	0.001 (0.001, 0.002)	0.01 (0.009, 0.011)	0.049 (0.047, 0.053)	0.099 (0.094, 0.104)
NBMM-AGQ	0.014 (0.012, 0.016)	0.046 (0.044, 0.049)	0.129 (0.125, 0.136)	0.206 (0.199, 0.213)	0.02 (0.018, 0.021)	0.05 (0.045, 0.054)	0.116 (0.11, 0.124)	0.18 (0.175, 0.189)	0.036 (0.034, 0.038)	0.05 (0.047, 0.052)	0.094 (0.086, 0.097)	0.145 (0.136, 0.151)
NBMM-LP	0.012 (0.011, 0.012)	0.057 (0.054, 0.059)	0.168 (0.162, 0.176)	0.263 (0.262, 0.264)	0.004 (0.004, 0.005)	0.028 (0.026, 0.03)	0.104 (0.101, 0.107)	0.179 (0.175, 0.182)	0.003 (0.002, 0.003)	0.018 (0.018, 0.018)	0.071 (0.067, 0.075)	0.136 (0.131, 0.14)
NBMM-PL	0.001 (0, 0.002)	0.011 (0.01, 0.012)	0.06 (0.055, 0.063)	0.121 (0.119, 0.127)	0.001 (0.001, 0.002)	0.014 (0.013, 0.017)	0.066 (0.063, 0.07)	0.127 (0.121, 0.135)	0.002 (0.002, 0.003)	0.016 (0.014, 0.018)	0.067 (0.062, 0.071)	0.126 (0.121, 0.134)
rmRNAseq	0 (0, 0)	0 (0, 0)	0.002 (0.001, 0.003)	0.008 (0.006, 0.009)	0 (0, 0)	0 (0, 0)	0 (0, 0.001)	0.002 (0.001, 0.003)	0 (0, 0)	0 (0, 0)	0 (0, 0)	0.001 (0, 0.001)

2.3 False Discovery Rate

Table 3: False discovery rate across several testing thresholds for each sample size and hypothesis test. Mean (Range) acrossthe 10 simulated datasets.

	Difference Between Groups at Any Timepoint								
	n3			n5			n10		
	0.01	0.05	0.1	0.01	0.05	0.1	0.01	0.05	0.1
DESeq2	0.185 (0.173, 0.206)	0.265 (0.247, 0.28)	0.321 (0.305, 0.334)	0.135 (0.125, 0.146)	0.221 (0.209, 0.227)	0.28 (0.271, 0.288)	0.105 (0.091, 0.112)	0.187 (0.174, 0.194)	0.244 (0.234, 0.25)
edgeR	0.154 (0.146, 0.164)	0.221 (0.206, 0.231)	0.266 (0.256, 0.282)	0.115 (0.105, 0.126)	0.19 (0.181, 0.195)	0.243 (0.235, 0.251)	0.103 (0.089, 0.108)	0.178 (0.161, 0.184)	0.231 (0.221, 0.238)
GEE	0.713 (0.708, 0.722)	0.731 (0.728, 0.737)	0.74 (0.738, 0.744)	0.356 (0.348, 0.365)	0.452 (0.445, 0.458)	0.507 (0.501, 0.513)	0.088 (0.076, 0.099)	0.185 (0.172, 0.2)	0.257 (0.247, 0.274)
limma	0.015 (0.006, 0.023)	0.055 (0.04, 0.072)	0.102 (0.073, 0.129)	0.019 (0.013, 0.031)	0.068 (0.047, 0.099)	0.122 (0.086, 0.177)	0.021 (0.013, 0.044)	0.076 (0.053, 0.138)	0.132 (0.091, 0.216)
LMM	0 (0, 0)	0.008 (0, 0.013)	0.023 (0.009, 0.029)	0.003 (0.002, 0.006)	0.025 (0.021, 0.033)	0.054 (0.048, 0.059)	0.006 (0.005, 0.008)	0.034 (0.027, 0.04)	0.07 (0.063, 0.08)
NBMM-AGQ	0.202 (0.167, 0.233)	0.32 (0.3, 0.338)	0.392 (0.381, 0.406)	0.232 (0.209, 0.243)	0.276 (0.257, 0.293)	0.32 (0.308, 0.339)	0.167 (0.161, 0.179)	0.187 (0.18, 0.195)	0.221 (0.213, 0.228)
NBMM-LP	0.095 (0.079, 0.108)	0.243 (0.23, 0.256)	0.352 (0.342, 0.365)	0.037 (0.033, 0.041)	0.121 (0.116, 0.124)	0.203 (0.2, 0.207)	0.018 (0.018, 0.018)	0.074 (0.069, 0.08)	0.129 (0.122, 0.137)
NBMM-PL	0.002 (0, 0.019)	0.012 (0.006, 0.026)	0.033 (0.017, 0.044)	0.006 (0.003, 0.01)	0.036 (0.028, 0.048)	0.077 (0.069, 0.088)	0.01 (0.006, 0.013)	0.05 (0.044, 0.059)	0.098 (0.091, 0.111)
rmRNAseq	0 (0, 0)	0.011 (0.005, 0.018)	0.025 (0.017, 0.03)	0 (0, 0.001)	0.001 (0, 0.003)	0.004 (0.002, 0.006)	0 (0, 0)	0 (0, 0.001)	0.001 (0, 0.001)

	Difference Between Any Timepoints in the Treatment Group								
	n3			n5			n10		
	0.01	0.05	0.1	0.01	0.05	0.1	0.01	0.05	0.1
DESeq2	0.013 (0.004, 0.019)	0.035 (0.03, 0.04)	0.057 (0.049, 0.062)	0.006 (0.002, 0.01)	0.02 (0.012, 0.022)	0.038 (0.031, 0.042)	0.005 (0.001, 0.009)	0.017 (0.013, 0.02)	0.031 (0.025, 0.034)
DESeq2*	0.046 (0.038, 0.051)	0.106 (0.098, 0.113)	0.155 (0.144, 0.164)	0.023 (0.019, 0.028)	0.072 (0.061, 0.08)	0.118 (0.112, 0.132)	0.014 (0.011, 0.018)	0.052 (0.044, 0.06)	0.095 (0.083, 0.107)
edgeR	0.01 (0.004, 0.016)	0.028 (0.02, 0.035)	0.045 (0.035, 0.053)	0.005 (0.002, 0.008)	0.016 (0.011, 0.019)	0.032 (0.024, 0.036)	0.004 (0.001, 0.007)	0.015 (0.012, 0.018)	0.028 (0.023, 0.033)
edgeR*	0.035 (0.025, 0.04)	0.08 (0.072, 0.091)	0.12 (0.112, 0.129)	0.018 (0.012, 0.023)	0.058 (0.049, 0.067)	0.096 (0.091, 0.113)	0.012 (0.01, 0.016)	0.044 (0.037, 0.051)	0.082 (0.071, 0.091)
GEE	0.58 (0.574, 0.586)	0.62 (0.615, 0.626)	0.643 (0.639, 0.65)	0.297 (0.291, 0.304)	0.39 (0.386, 0.397)	0.445 (0.433, 0.451)	0.125 (0.111, 0.134)	0.209 (0.197, 0.223)	0.273 (0.262, 0.283)
limma	0.009 (0.002, 0.019)	0.046 (0.034, 0.068)	0.091 (0.069, 0.124)	0.016 (0.007, 0.028)	0.068 (0.043, 0.118)	0.127 (0.09, 0.204)	0.032 (0.013, 0.088)	0.111 (0.049, 0.228)	0.182 (0.1, 0.325)
LMM	0.01 (0.006, 0.014)	0.044 (0.036, 0.05)	0.088 (0.079, 0.099)	0.008 (0.005, 0.012)	0.043 (0.035, 0.054)	0.084 (0.078, 0.096)	0.009 (0.006, 0.01)	0.041 (0.034, 0.047)	0.081 (0.069, 0.089)
NBMM-AGQ	0.238 (0.216, 0.253)	0.347 (0.335, 0.369)	0.417 (0.407, 0.438)	0.189 (0.17, 0.205)	0.243 (0.218, 0.256)	0.296 (0.28, 0.307)	0.095 (0.088, 0.102)	0.132 (0.127, 0.142)	0.179 (0.171, 0.192)
NBMM-LP	0.083 (0.08, 0.089)	0.217 (0.202, 0.225)	0.318 (0.308, 0.329)	0.03 (0.025, 0.037)	0.111 (0.106, 0.12)	0.185 (0.179, 0.191)	0.016 (0.013, 0.019)	0.064 (0.058, 0.07)	0.116 (0.107, 0.124)
NBMM-PL	0.014 (0.009, 0.019)	0.058 (0.051, 0.066)	0.115 (0.1, 0.129)	0.015 (0.012, 0.018)	0.065 (0.052, 0.08)	0.118 (0.109, 0.135)	0.016 (0.012, 0.021)	0.06 (0.051, 0.067)	0.11 (0.099, 0.118)
rmRNAseq	0 (0, 0)	0 (0, 0.003)	0.001 (0, 0.009)	0 (0, 0)	0 (0, 0.001)	0 (0, 0.002)	0 (0, 0)	0 (0, 0)	0 (0, 0)

	Any Significant Interaction Effect								
	n3			n5			n10		
	0.01	0.05	0.1	0.01	0.05	0.1	0.01	0.05	0.1
DESeq2	0.019 (0.011, 0.029)	0.047 (0.035, 0.061)	0.069 (0.062, 0.076)	0.007 (0.004, 0.01)	0.022 (0.016, 0.027)	0.039 (0.033, 0.049)	0.004 (0.002, 0.006)	0.016 (0.013, 0.019)	0.03 (0.026, 0.035)
DESeq2*	0.064 (0.057, 0.07)	0.129 (0.123, 0.144)	0.179 (0.168, 0.187)	0.028 (0.024, 0.036)	0.077 (0.065, 0.089)	0.123 (0.11, 0.137)	0.014 (0.012, 0.018)	0.055 (0.049, 0.062)	0.101 (0.092, 0.11)
edgeR	0.022 (0, 0.049)	0.046 (0.028, 0.071)	0.064 (0.048, 0.081)	0.006 (0.004, 0.009)	0.019 (0.012, 0.027)	0.033 (0.024, 0.043)	0.003 (0.001, 0.006)	0.015 (0.01, 0.018)	0.028 (0.026, 0.032)
edgeR*	0.056 (0.049, 0.068)	0.107 (0.098, 0.115)	0.148 (0.141, 0.153)	0.021 (0.017, 0.026)	0.061 (0.051, 0.072)	0.099 (0.091, 0.11)	0.012 (0.008, 0.014)	0.046 (0.04, 0.052)	0.088 (0.081, 0.094)
GEE	0.576 (0.568, 0.589)	0.615 (0.609, 0.624)	0.638 (0.633, 0.649)	0.227 (0.218, 0.235)	0.326 (0.317, 0.33)	0.39 (0.384, 0.399)	0.059 (0.049, 0.07)	0.136 (0.125, 0.148)	0.201 (0.186, 0.214)
limma	0.009 (0.003, 0.018)	0.043 (0.026, 0.055)	0.084 (0.066, 0.101)	0.011 (0.005, 0.022)	0.054 (0.036, 0.096)	0.103 (0.072, 0.176)	0.016 (0.006, 0.026)	0.067 (0.047, 0.097)	0.121 (0.082, 0.17)
LMM	0.011 (0.002, 0.022)	0.046 (0.039, 0.058)	0.091 (0.078, 0.107)	0.008 (0.005, 0.013)	0.042 (0.037, 0.049)	0.081 (0.071, 0.095)	0.008 (0.005, 0.01)	0.041 (0.035, 0.05)	0.084 (0.075, 0.093)
NBMM-AGQ	0.25 (0.214, 0.275)	0.369 (0.349, 0.389)	0.445 (0.432, 0.456)	0.203 (0.188, 0.222)	0.254 (0.236, 0.274)	0.304 (0.286, 0.321)	0.098 (0.09, 0.108)	0.138 (0.125, 0.145)	0.186 (0.177, 0.193)
NBMM-LP	0.1 (0.093, 0.112)	0.239 (0.23, 0.246)	0.346 (0.335, 0.356)	0.034 (0.031, 0.036)	0.12 (0.115, 0.128)	0.198 (0.192, 0.203)	0.015 (0.012, 0.018)	0.072 (0.064, 0.079)	0.13 (0.123, 0.136)
NBMM-PL	0.014 (0.004, 0.02)	0.061 (0.054, 0.071)	0.122 (0.111, 0.137)	0.017 (0.009, 0.022)	0.065 (0.059, 0.072)	0.12 (0.106, 0.137)	0.016 (0.012, 0.019)	0.064 (0.055, 0.071)	0.117 (0.106, 0.129)
rmRNAseq	0 (0, 0)	0.009 (0, 0.024)	0.018 (0.013, 0.026)	0 (0, 0)	0 (0, 0.001)	0.001 (0, 0.003)	0 (0, 0)	0 (0, 0.001)	0 (0, 0.001)

Table 3: False discovery rate across several testing thresholds for each sample size and hypothesis test. Mean (Range) acrossthe 10 simulated datasets (continued).

	Any Significant Coefficient								
	n3			n5			n10		
	0.01	0.05	0.1	0.01	0.05	0.1	0.01	0.05	0.1
DESeq2	0.144 (0.127, 0.161)	0.215 (0.202, 0.226)	0.262 (0.247, 0.274)	0.097 (0.088, 0.104)	0.162 (0.153, 0.171)	0.211 (0.195, 0.219)	0.073 (0.062, 0.079)	0.132 (0.119, 0.14)	0.177 (0.165, 0.183)
DESeq2*	0.084 (0.073, 0.096)	0.156 (0.142, 0.164)	0.213 (0.203, 0.221)	0.036 (0.031, 0.044)	0.096 (0.091, 0.104)	0.148 (0.129, 0.159)	0.018 (0.015, 0.019)	0.064 (0.059, 0.07)	0.112 (0.105, 0.118)
edgeR	0.119 (0.109, 0.13)	0.177 (0.17, 0.183)	0.213 (0.201, 0.221)	0.083 (0.075, 0.091)	0.138 (0.13, 0.144)	0.179 (0.174, 0.183)	0.074 (0.06, 0.082)	0.127 (0.112, 0.134)	0.167 (0.151, 0.174)
edgeR*	0.066 (0.058, 0.076)	0.121 (0.11, 0.129)	0.167 (0.159, 0.174)	0.028 (0.021, 0.036)	0.075 (0.069, 0.083)	0.115 (0.102, 0.125)	0.015 (0.014, 0.016)	0.053 (0.044, 0.057)	0.093 (0.086, 0.101)
GEE	0.735 (0.732, 0.739)	0.75 (0.747, 0.752)	0.757 (0.755, 0.759)	0.471 (0.466, 0.48)	0.553 (0.55, 0.559)	0.595 (0.591, 0.597)	0.197 (0.18, 0.205)	0.304 (0.284, 0.314)	0.377 (0.367, 0.389)
limma	0.011 (0.008, 0.022)	0.047 (0.038, 0.068)	0.09 (0.074, 0.105)	0.016 (0.013, 0.021)	0.064 (0.047, 0.087)	0.114 (0.087, 0.159)	0.024 (0.012, 0.053)	0.085 (0.051, 0.166)	0.147 (0.094, 0.262)
LMM	0.006 (0.002, 0.009)	0.031 (0.024, 0.039)	0.065 (0.057, 0.072)	0.005 (0.004, 0.008)	0.035 (0.031, 0.042)	0.073 (0.066, 0.087)	0.008 (0.005, 0.011)	0.039 (0.035, 0.043)	0.078 (0.073, 0.083)
NBMM-AGQ	0.171 (0.15, 0.208)	0.246 (0.232, 0.267)	0.319 (0.305, 0.326)	0.145 (0.129, 0.154)	0.223 (0.213, 0.238)	0.28 (0.265, 0.305)	0.164 (0.155, 0.173)	0.193 (0.183, 0.203)	0.229 (0.215, 0.238)
NBMM-LP	0.083 (0.08, 0.087)	0.228 (0.215, 0.238)	0.34 (0.333, 0.351)	0.032 (0.029, 0.038)	0.112 (0.106, 0.118)	0.189 (0.18, 0.194)	0.018 (0.016, 0.02)	0.07 (0.07, 0.071)	0.124 (0.119, 0.129)
NBMM-PL	0.008 (0.001, 0.014)	0.042 (0.036, 0.049)	0.089 (0.082, 0.102)	0.013 (0.008, 0.018)	0.058 (0.051, 0.073)	0.11 (0.1, 0.129)	0.017 (0.013, 0.021)	0.065 (0.058, 0.071)	0.12 (0.109, 0.129)
rmRNAseq	0 (0, 0)	0 (0, 0)	0 (0, 0)	0 (0, 0)	0 (0, 0)	0 (0, 0.001)	0 (0, 0)	0 (0, 0)	0 (0, 0)

2.4 Power

Table 4: Power across several testing thresholds for each sample size and hypothesis test. Mean (Range) acrossthe 10 simulated datasets.

	Difference Between Groups at Any Timepoint								
	n3			n5			n10		
	0.01	0.05	0.1	0.01	0.05	0.1	0.01	0.05	0.1
DESeq2	0.509 (0.495, 0.524)	0.612 (0.597, 0.624)	0.667 (0.655, 0.679)	0.67 (0.648, 0.687)	0.746 (0.725, 0.762)	0.786 (0.763, 0.8)	0.828 (0.811, 0.833)	0.873 (0.862, 0.88)	0.894 (0.884, 0.902)
edgeR	0.459 (0.439, 0.476)	0.579 (0.564, 0.597)	0.638 (0.624, 0.648)	0.66 (0.64, 0.675)	0.739 (0.716, 0.757)	0.78 (0.757, 0.798)	0.83 (0.815, 0.836)	0.873 (0.865, 0.878)	0.895 (0.885, 0.902)
GEE	0.795 (0.784, 0.81)	0.849 (0.837, 0.858)	0.879 (0.864, 0.887)	0.712 (0.689, 0.734)	0.786 (0.773, 0.797)	0.824 (0.813, 0.835)	0.801 (0.79, 0.819)	0.852 (0.841, 0.861)	0.877 (0.868, 0.886)
limma	0.239 (0.205, 0.265)	0.403 (0.373, 0.425)	0.488 (0.462, 0.5)	0.531 (0.511, 0.549)	0.64 (0.618, 0.661)	0.691 (0.673, 0.706)	0.757 (0.744, 0.766)	0.813 (0.803, 0.823)	0.84 (0.831, 0.848)
LMM	0.008 (0, 0.025)	0.182 (0.159, 0.206)	0.311 (0.28, 0.329)	0.482 (0.451, 0.499)	0.626 (0.601, 0.644)	0.691 (0.666, 0.712)	0.786 (0.777, 0.809)	0.843 (0.831, 0.856)	0.868 (0.861, 0.877)
NBMM-AGQ	0.22 (0.205, 0.229)	0.37 (0.354, 0.39)	0.463 (0.438, 0.479)	0.389 (0.37, 0.412)	0.523 (0.501, 0.54)	0.59 (0.57, 0.608)	0.622 (0.598, 0.633)	0.706 (0.679, 0.719)	0.743 (0.717, 0.756)
NBMM-LP	0.542 (0.526, 0.56)	0.684 (0.671, 0.692)	0.753 (0.748, 0.761)	0.674 (0.652, 0.696)	0.77 (0.764, 0.778)	0.811 (0.803, 0.824)	0.824 (0.823, 0.824)	0.871 (0.871, 0.872)	0.894 (0.89, 0.898)
NBMM-PL	0.013 (0, 0.025)	0.199 (0.173, 0.22)	0.337 (0.304, 0.357)	0.506 (0.484, 0.526)	0.659 (0.635, 0.679)	0.728 (0.704, 0.743)	0.817 (0.805, 0.833)	0.875 (0.867, 0.883)	0.899 (0.888, 0.912)
rmRNAseq	0.008 (0, 0.044)	0.212 (0.14, 0.258)	0.322 (0.26, 0.366)	0.244 (0.194, 0.307)	0.365 (0.312, 0.417)	0.428 (0.376, 0.488)	0.473 (0.429, 0.529)	0.589 (0.538, 0.64)	0.648 (0.602, 0.686)

	Difference Between Any Timepoints in the Treatment Group								
	n3			n5			n10		
	0.01	0.05	0.1	0.01	0.05	0.1	0.01	0.05	0.1
DESeq2	0.39 (0.379, 0.404)	0.492 (0.469, 0.507)	0.55 (0.533, 0.569)	0.578 (0.554, 0.6)	0.668 (0.651, 0.689)	0.712 (0.696, 0.726)	0.778 (0.764, 0.795)	0.837 (0.826, 0.851)	0.864 (0.85, 0.877)
DESeq2*	0.602 (0.588, 0.626)	0.69 (0.677, 0.71)	0.737 (0.726, 0.754)	0.742 (0.726, 0.758)	0.804 (0.793, 0.813)	0.833 (0.822, 0.845)	0.861 (0.851, 0.867)	0.898 (0.889, 0.905)	0.914 (0.905, 0.924)
edgeR	0.315 (0.305, 0.327)	0.444 (0.417, 0.46)	0.511 (0.487, 0.525)	0.561 (0.538, 0.586)	0.659 (0.641, 0.68)	0.706 (0.69, 0.72)	0.777 (0.763, 0.794)	0.837 (0.826, 0.85)	0.863 (0.85, 0.876)
edgeR*	0.588 (0.575, 0.613)	0.685 (0.67, 0.705)	0.734 (0.724, 0.754)	0.743 (0.73, 0.757)	0.805 (0.794, 0.815)	0.834 (0.822, 0.846)	0.862 (0.852, 0.867)	0.899 (0.89, 0.905)	0.914 (0.905, 0.924)
GEE	0.798 (0.788, 0.815)	0.853 (0.844, 0.869)	0.881 (0.867, 0.897)	0.794 (0.784, 0.804)	0.851 (0.844, 0.865)	0.88 (0.867, 0.896)	0.876 (0.865, 0.88)	0.912 (0.901, 0.916)	0.929 (0.92, 0.933)
limma	0.411 (0.353, 0.436)	0.556 (0.513, 0.588)	0.624 (0.593, 0.657)	0.651 (0.634, 0.672)	0.738 (0.718, 0.757)	0.779 (0.762, 0.796)	0.824 (0.81, 0.831)	0.867 (0.857, 0.873)	0.887 (0.88, 0.896)
LMM	0.418 (0.402, 0.44)	0.583 (0.571, 0.605)	0.654 (0.641, 0.673)	0.68 (0.666, 0.693)	0.767 (0.751, 0.788)	0.807 (0.796, 0.822)	0.849 (0.842, 0.857)	0.889 (0.878, 0.896)	0.905 (0.896, 0.911)
NBMM-AGQ	0.286 (0.265, 0.304)	0.454 (0.441, 0.473)	0.551 (0.54, 0.575)	0.472 (0.45, 0.494)	0.602 (0.59, 0.62)	0.665 (0.651, 0.682)	0.697 (0.675, 0.708)	0.771 (0.739, 0.786)	0.81 (0.781, 0.821)
NBMM-LP	0.655 (0.642, 0.665)	0.77 (0.766, 0.777)	0.821 (0.818, 0.825)	0.754 (0.749, 0.763)	0.827 (0.82, 0.832)	0.86 (0.855, 0.865)	0.857 (0.856, 0.858)	0.898 (0.895, 0.9)	0.917 (0.917, 0.917)
NBMM-PL	0.437 (0.421, 0.458)	0.611 (0.6, 0.627)	0.691 (0.681, 0.708)	0.716 (0.701, 0.728)	0.808 (0.788, 0.826)	0.848 (0.831, 0.867)	0.883 (0.875, 0.892)	0.922 (0.911, 0.93)	0.939 (0.932, 0.945)
rmRNAseq	0.014 (0, 0.042)	0.092 (0.019, 0.146)	0.176 (0.078, 0.244)	0.169 (0.124, 0.243)	0.317 (0.271, 0.381)	0.401 (0.358, 0.473)	0.495 (0.444, 0.56)	0.624 (0.589, 0.667)	0.683 (0.654, 0.72)

Table 4: Power across several testing thresholds for each sample size and hypothesis test. Mean (Range) acrossthe 10 simulated datasets (continued).

	Any Significant Interaction Effect								
	n3			n5			n10		
	0.01	0.05	0.1	0.01	0.05	0.1	0.01	0.05	0.1
DESeq2	0.149 (0.142, 0.161)	0.234 (0.224, 0.245)	0.288 (0.276, 0.303)	0.316 (0.302, 0.332)	0.421 (0.401, 0.437)	0.479 (0.456, 0.496)	0.577 (0.555, 0.594)	0.67 (0.652, 0.691)	0.715 (0.7, 0.731)
DESeq2*	0.363 (0.352, 0.383)	0.479 (0.467, 0.505)	0.54 (0.524, 0.557)	0.545 (0.53, 0.558)	0.641 (0.622, 0.65)	0.692 (0.673, 0.704)	0.748 (0.742, 0.765)	0.805 (0.795, 0.82)	0.834 (0.825, 0.844)
edgeR	0.019 (0.009, 0.033)	0.092 (0.072, 0.104)	0.16 (0.134, 0.18)	0.263 (0.254, 0.276)	0.383 (0.364, 0.401)	0.448 (0.426, 0.469)	0.569 (0.549, 0.588)	0.665 (0.647, 0.686)	0.712 (0.697, 0.726)
edgeR*	0.288 (0.266, 0.316)	0.429 (0.417, 0.451)	0.504 (0.493, 0.523)	0.534 (0.523, 0.546)	0.635 (0.617, 0.644)	0.688 (0.668, 0.702)	0.749 (0.742, 0.764)	0.806 (0.795, 0.82)	0.835 (0.827, 0.846)
GEE	0.58 (0.563, 0.601)	0.67 (0.661, 0.685)	0.721 (0.706, 0.732)	0.573 (0.551, 0.587)	0.665 (0.651, 0.677)	0.716 (0.702, 0.729)	0.718 (0.702, 0.743)	0.782 (0.769, 0.805)	0.815 (0.804, 0.83)
limma	0.124 (0.083, 0.152)	0.286 (0.233, 0.304)	0.378 (0.331, 0.4)	0.426 (0.404, 0.444)	0.547 (0.525, 0.562)	0.611 (0.591, 0.624)	0.687 (0.672, 0.702)	0.76 (0.746, 0.774)	0.795 (0.784, 0.807)
LMM	0.173 (0.161, 0.189)	0.329 (0.311, 0.355)	0.424 (0.405, 0.446)	0.461 (0.439, 0.487)	0.587 (0.565, 0.6)	0.649 (0.624, 0.662)	0.726 (0.714, 0.747)	0.791 (0.777, 0.808)	0.822 (0.811, 0.836)
NBMM-AGQ	0.277 (0.263, 0.293)	0.405 (0.392, 0.418)	0.487 (0.474, 0.503)	0.416 (0.403, 0.423)	0.54 (0.524, 0.555)	0.612 (0.597, 0.63)	0.656 (0.641, 0.667)	0.741 (0.724, 0.75)	0.782 (0.763, 0.792)
NBMM-LP	0.429 (0.415, 0.44)	0.593 (0.58, 0.599)	0.677 (0.675, 0.679)	0.559 (0.547, 0.568)	0.678 (0.668, 0.687)	0.735 (0.726, 0.744)	0.735 (0.73, 0.739)	0.8 (0.797, 0.803)	0.833 (0.829, 0.838)
NBMM-PL	0.181 (0.169, 0.195)	0.354 (0.337, 0.38)	0.458 (0.445, 0.477)	0.487 (0.467, 0.514)	0.625 (0.604, 0.635)	0.693 (0.666, 0.712)	0.759 (0.747, 0.78)	0.829 (0.814, 0.846)	0.861 (0.851, 0.873)
rmRNAseq	0.03 (0.004, 0.061)	0.102 (0.042, 0.166)	0.17 (0.094, 0.238)	0.129 (0.1, 0.185)	0.21 (0.175, 0.269)	0.262 (0.226, 0.314)	0.335 (0.277, 0.403)	0.46 (0.409, 0.517)	0.526 (0.478, 0.578)

	Any Significant Coefficient								
	n3			n5			n10		
	0.01	0.05	0.1	0.01	0.05	0.1	0.01	0.05	0.1
DESeq2	0.551 (0.538, 0.569)	0.641 (0.622, 0.66)	0.687 (0.672, 0.706)	0.706 (0.689, 0.716)	0.774 (0.752, 0.784)	0.805 (0.78, 0.816)	0.852 (0.837, 0.858)	0.895 (0.883, 0.9)	0.913 (0.902, 0.919)
DESeq2*	0.54 (0.532, 0.564)	0.635 (0.62, 0.655)	0.684 (0.672, 0.702)	0.693 (0.681, 0.706)	0.764 (0.753, 0.777)	0.797 (0.786, 0.808)	0.838 (0.83, 0.845)	0.874 (0.865, 0.882)	0.891 (0.88, 0.898)
edgeR	0.505 (0.491, 0.528)	0.613 (0.589, 0.635)	0.664 (0.645, 0.686)	0.698 (0.682, 0.709)	0.768 (0.747, 0.779)	0.802 (0.777, 0.813)	0.853 (0.839, 0.858)	0.895 (0.884, 0.902)	0.914 (0.904, 0.92)
edgeR*	0.51 (0.492, 0.527)	0.619 (0.6, 0.64)	0.674 (0.661, 0.693)	0.692 (0.681, 0.706)	0.764 (0.751, 0.777)	0.798 (0.787, 0.811)	0.839 (0.83, 0.846)	0.875 (0.866, 0.883)	0.892 (0.881, 0.898)
GEE	0.933 (0.924, 0.948)	0.958 (0.953, 0.965)	0.969 (0.963, 0.974)	0.862 (0.855, 0.873)	0.908 (0.898, 0.917)	0.93 (0.922, 0.939)	0.901 (0.89, 0.907)	0.934 (0.927, 0.941)	0.951 (0.944, 0.957)
limma	0.327 (0.288, 0.353)	0.484 (0.447, 0.505)	0.562 (0.531, 0.579)	0.615 (0.594, 0.631)	0.706 (0.69, 0.726)	0.75 (0.733, 0.77)	0.813 (0.803, 0.826)	0.854 (0.849, 0.859)	0.874 (0.869, 0.878)
LMM	0.25 (0.236, 0.266)	0.44 (0.411, 0.456)	0.535 (0.504, 0.557)	0.625 (0.608, 0.644)	0.726 (0.705, 0.742)	0.774 (0.759, 0.788)	0.843 (0.837, 0.852)	0.883 (0.874, 0.888)	0.9 (0.893, 0.908)
NBMM-AGQ	0.201 (0.176, 0.223)	0.384 (0.366, 0.405)	0.492 (0.47, 0.509)	0.486 (0.463, 0.51)	0.628 (0.608, 0.652)	0.692 (0.678, 0.715)	0.772 (0.756, 0.781)	0.835 (0.818, 0.844)	0.862 (0.85, 0.869)
NBMM-LP	0.607 (0.587, 0.623)	0.738 (0.73, 0.748)	0.8 (0.79, 0.811)	0.743 (0.738, 0.753)	0.818 (0.812, 0.825)	0.852 (0.843, 0.856)	0.867 (0.867, 0.867)	0.902 (0.9, 0.904)	0.917 (0.915, 0.92)
NBMM-PL	0.262 (0.252, 0.27)	0.464 (0.438, 0.479)	0.57 (0.547, 0.584)	0.655 (0.641, 0.669)	0.769 (0.747, 0.784)	0.818 (0.797, 0.832)	0.877 (0.867, 0.885)	0.92 (0.911, 0.927)	0.937 (0.929, 0.943)
rmRNAseq	0 (0, 0.002)	0.006 (0, 0.023)	0.026 (0, 0.082)	0.073 (0.03, 0.146)	0.218 (0.169, 0.288)	0.299 (0.249, 0.362)	0.421 (0.372, 0.487)	0.56 (0.515, 0.614)	0.619 (0.568, 0.669)

2.5 P-value Distribution

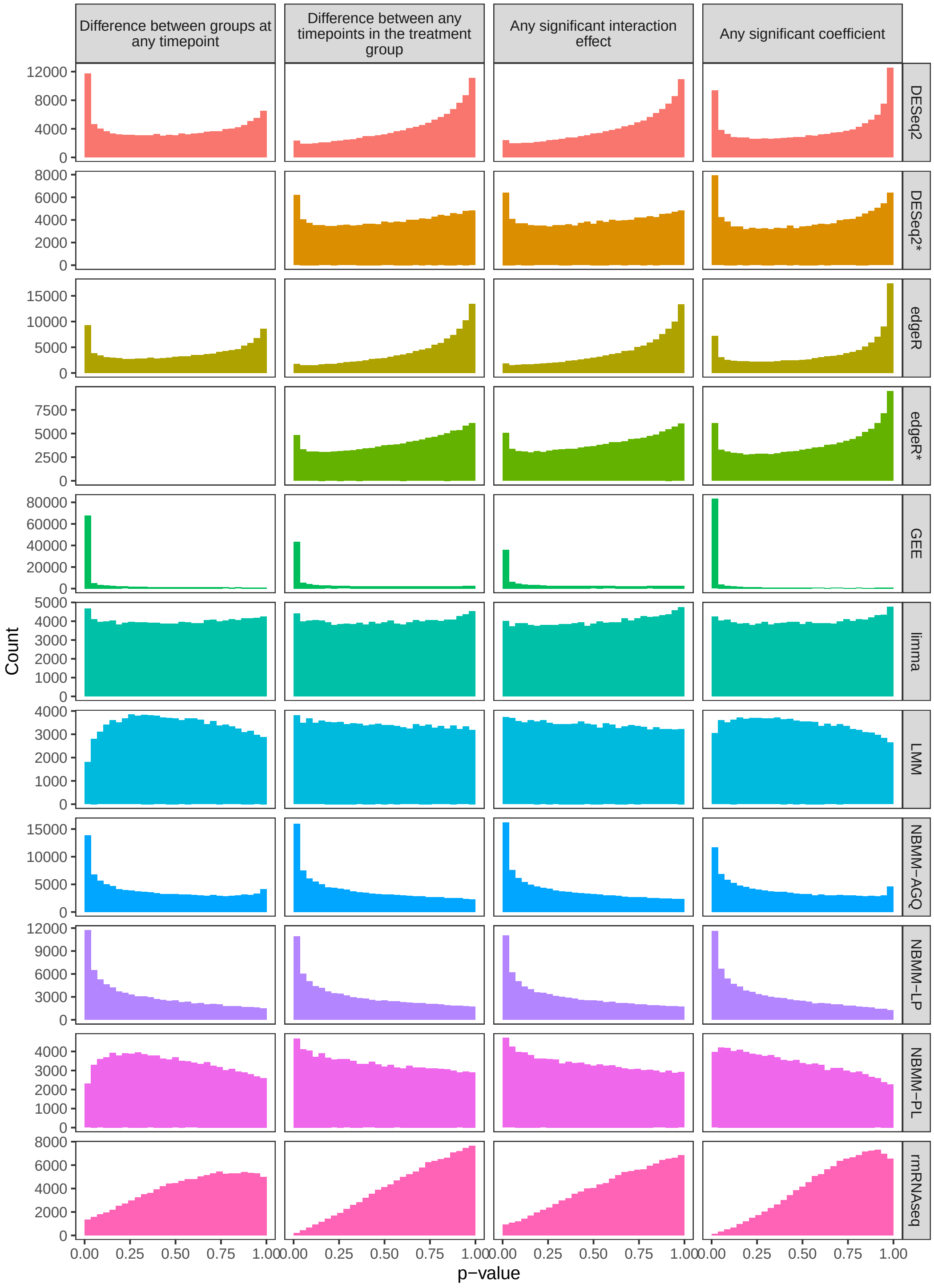

Figure 2: Histograms of p-values of null features by method for the four tests of interest in the $N = 3$ per group simulation datasets.

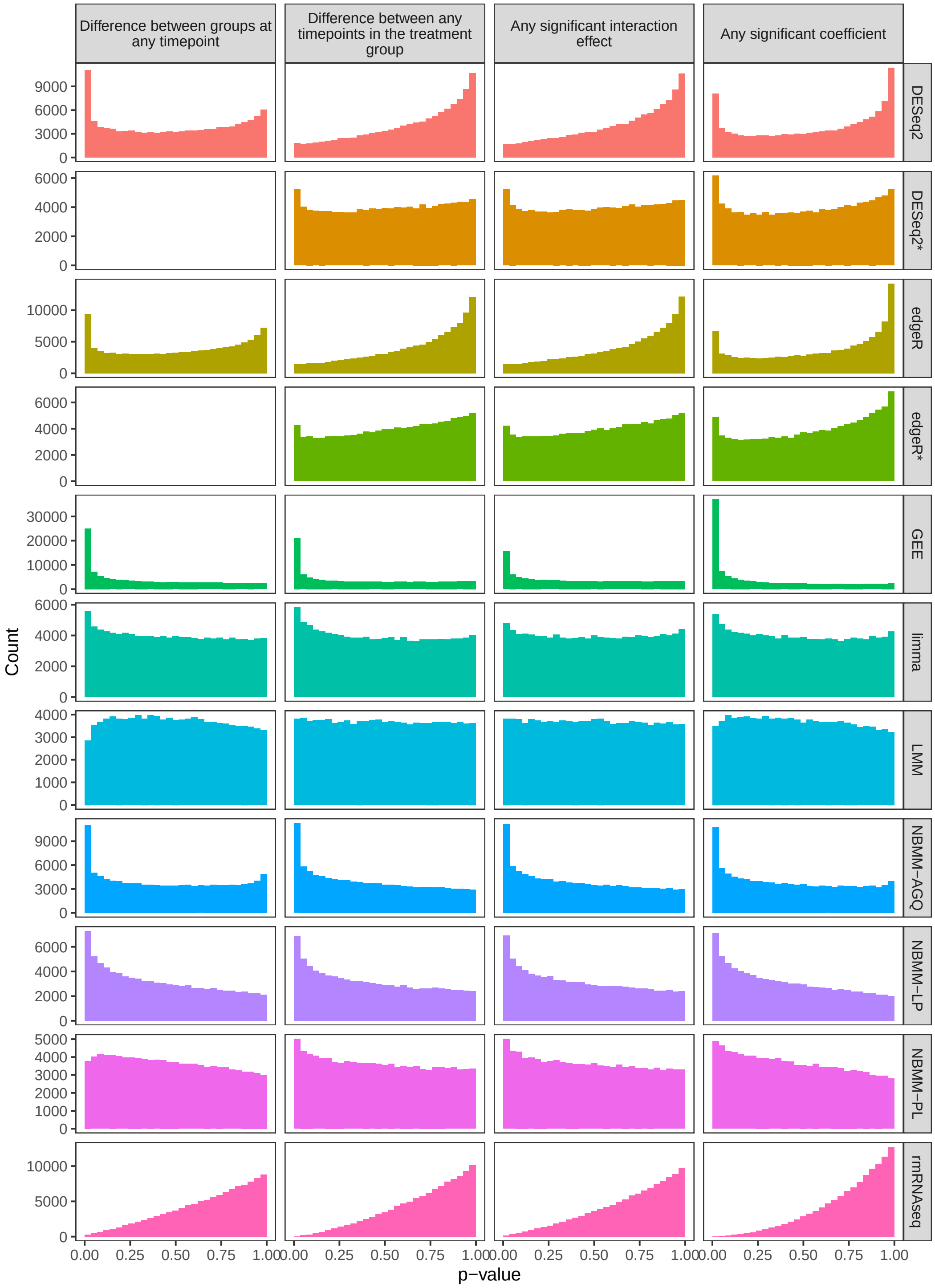

Figure 3: Histograms of p-values of null features by method for the four tests of interest in the $N = 5$ per group simulation datasets.

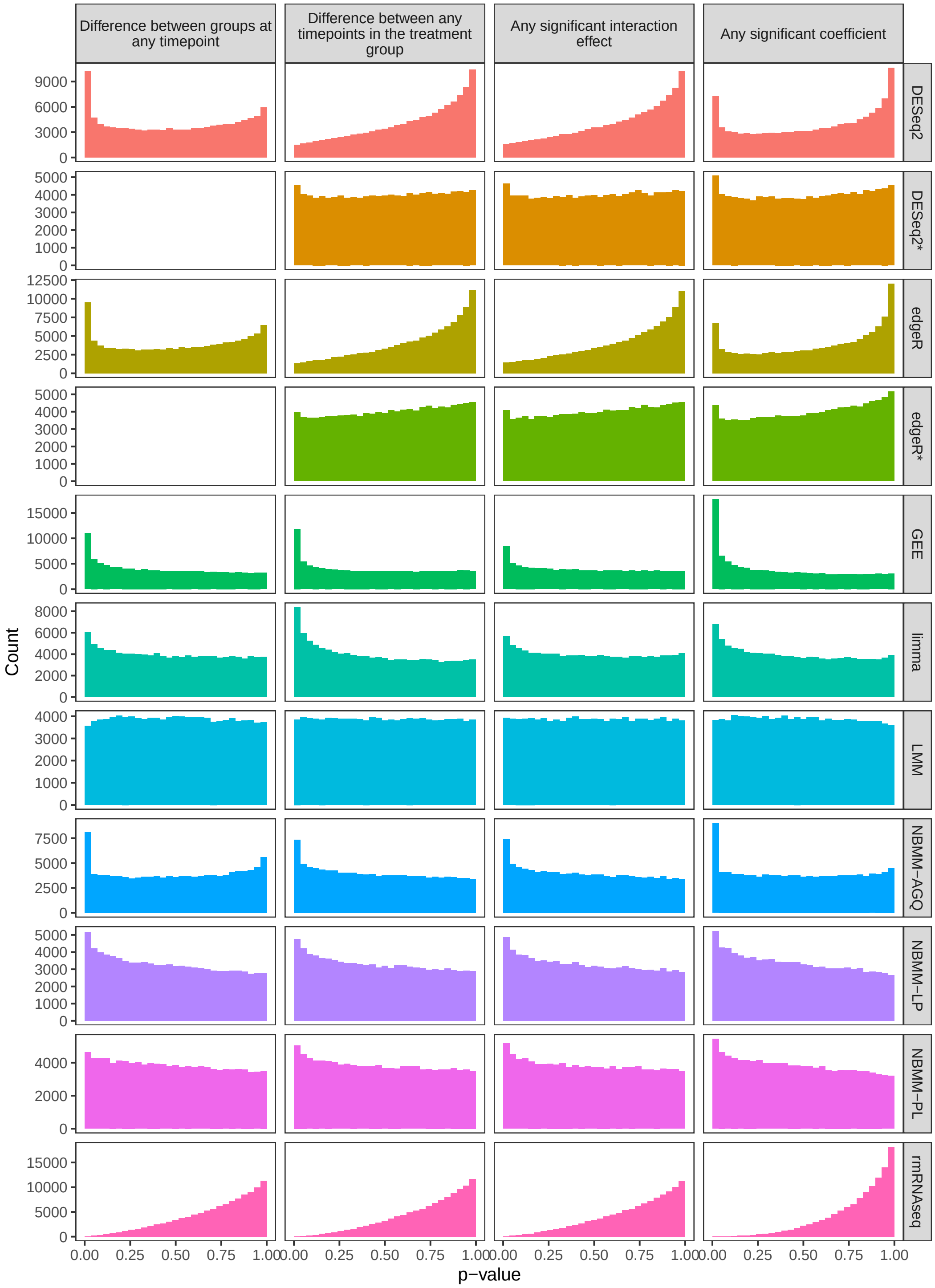

Figure 4: Histograms of p-values of null features by method for the four tests of interest in the $N = 10$ per group simulation datasets.

3 Additional Application Results

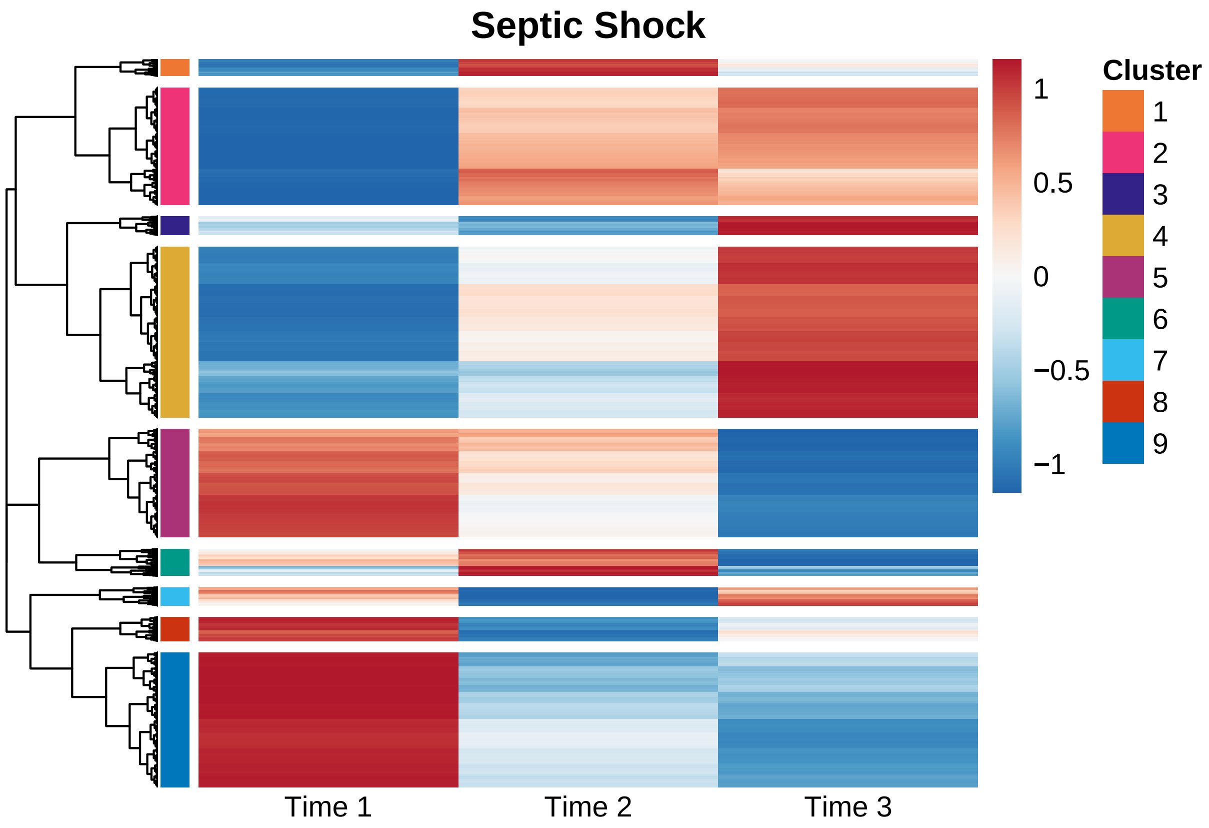

Figure 5: Heatmap of predicted gene expression (row scaled) across the three study timepoints for genes that were significant in a test for differential expression between any two timepoints in the SS group. Predicted values and significance results came from the LMM analysis. Genes are clustered using a correlation distance metric and complete linkage clustering methods and are split into seven clusters indicated by the color bars along the rows.

Table 5: Functional enrichment analysis results for the SS group. The 25 GO terms with the smallest Benjamini Hochberg (BH) adjusted p-values were selected for each cluster. The lists were then reduced to include only the most specific subclass for each ontology. All GO terms had a BH adjusted p-value < 0.01.

GO Term	Description	# Genes in Set	# Genes in Cluster	# Expected	Fold Enrichment
**Cluster 2**					
GO:0006614	SRP-dependent cotranslational protein ta...	97	59	10.14	5.82
GO:0000184	nuclear-transcribed mRNA catabolic proce...	119	61	12.44	4.90
GO:0006364	rRNA processing	224	78	23.42	3.33
GO:0006413	translational initiation	184	69	19.23	3.59
GO:0019083	viral transcription	175	63	18.29	3.44
GO:0070125	mitochondrial translational elongation	86	38	8.99	4.23
GO:0042273	ribosomal large subunit biogenesis	71	34	7.42	4.58
GO:0070126	mitochondrial translational termination	87	37	9.09	4.07
GO:0042255	ribosome assembly	61	30	6.38	4.70
GO:0006400	tRNA modification	87	35	9.09	3.85
GO:0022618	ribonucleoprotein complex assembly	193	53	20.18	2.63
**Cluster 3**					
GO:0060986	endocrine hormone secretion	22	6	0.40	15.00
**Cluster 4**					
GO:0006958	complement activation, classical pathway	83	53	12.88	4.11
GO:0002377	immunoglobulin production	128	61	19.86	3.07
GO:0050871	positive regulation of B cell activation	111	51	17.22	2.96
GO:0006910	phagocytosis, recognition	54	33	8.38	3.94
GO:0050853	B cell receptor signaling pathway	100	44	15.52	2.84
GO:0030449	regulation of complement activation	71	35	11.02	3.18
GO:0042742	defense response to bacterium	174	59	27.00	2.19
GO:0006911	phagocytosis, engulfment	87	34	13.50	2.52
GO:0045058	T cell selection	38	19	5.90	3.22
GO:0050900	leukocyte migration	357	90	55.40	1.62
GO:0038096	Fc-gamma receptor signaling pathway invo...	117	39	18.16	2.15
**Cluster 5**					
GO:0006954	inflammatory response	484	89	49.53	1.80
GO:0043312	neutrophil degranulation	453	81	46.36	1.75
GO:0002224	toll-like receptor signaling pathway	123	31	12.59	2.46
GO:0042116	macrophage activation	74	22	7.57	2.91
GO:0016236	macroautophagy	280	54	28.65	1.88
GO:0019320	hexose catabolic process	44	16	4.50	3.56
GO:0031349	positive regulation of defense response	268	52	27.43	1.90
GO:0006091	generation of precursor metabolites and ...	419	72	42.88	1.68
GO:0046034	ATP metabolic process	262	50	26.81	1.86
GO:0060334	regulation of interferon-gamma-mediated ...	21	10	2.15	4.65
GO:0071702	organic substance transport	1998	257	204.46	1.26
GO:2000377	regulation of reactive oxygen species me...	138	31	14.12	2.20
GO:0006508	proteolysis	1254	172	128.33	1.34
GO:0048193	Golgi vesicle transport	321	57	32.85	1.74
GO:0019674	NAD metabolic process	42	14	4.30	3.26
**Cluster 8**					
GO:0038128	ERBB2 signaling pathway	23	6	0.51	11.76
GO:0045022	early endosome to late endosome transpor...	38	7	0.85	8.24
GO:0031333	negative regulation of protein-containin...	104	11	2.33	4.72
GO:1903897	regulation of PERK-mediated unfolded pro...	9	4	0.20	20.00
GO:0032516	positive regulation of phosphoprotein ph...	19	5	0.43	11.63
GO:2001044	regulation of integrin-mediated signalin...	12	4	0.27	14.81
GO:1900102	negative regulation of endoplasmic retic...	13	4	0.29	13.79
GO:0019068	virion assembly	38	6	0.85	7.06
**Cluster 9**					
GO:0043312	neutrophil degranulation	453	124	58.13	2.13
GO:0060627	regulation of vesicle-mediated transport	360	92	46.19	1.99
GO:0051128	regulation of cellular component organiz...	1691	303	216.98	1.40
GO:0016050	vesicle organization	284	74	36.44	2.03
GO:0010256	endomembrane system organization	383	91	49.14	1.85
GO:0030168	platelet activation	125	40	16.04	2.49
GO:0048193	Golgi vesicle transport	321	77	41.19	1.87
GO:0050790	regulation of catalytic activity	1776	300	227.88	1.32
GO:0044087	regulation of cellular component biogene...	688	137	88.28	1.55
GO:0023052	signaling	3958	600	507.86	1.18
GO:0007154	cell communication	3975	602	510.05	1.18
GO:0051656	establishment of organelle localization	355	81	45.55	1.78

References

[1] Smyth, G.K.: limma: Linear Models for Microarray Data. In: Bioinformatics and Computational Biology Solutions Using R and Bioconductor, pp. 397–420. Springer, New York (2005)

[2] Ritchie, M.E., Phipson, B., Wu, D., Hu, Y., Law, C.W., Shi, W., Smyth, G.K.: Limma powers differential expression analyses for RNA-sequencing and microarray studies. Nucleic Acids Research **43**(7), 47 (2015). doi:10.1093/nar/gkv007

[3] Phipson, B., Lee, S., Majewski, I.J., Alexander, W.S., Smyth, G.K.: Robust hyperparameter estimation protects against hypervariable genes and improves power to detect differential expression. Annals of Applied Statistics **10**(2), 946–963 (2016). doi:10.1214/16-AOAS920. 1602.08678

[4] Robinson, M.D., Oshlack, A.: A scaling normalization method for differential expression analysis of RNA-seq data. Genome Biology **11**(3) (2010). doi:10.1186/gb-2010-11-3-r25

[5] McCarthy, D.J., Chen, Y., Smyth, G.K.: Differential expression analysis of multifactor RNA-Seq experiments with respect to biological variation. Nucleic Acids Research **40**(10), 4288–4297 (2012). doi:10.1093/nar/gks042

[6] Anders, S., Huber, W.: Differential expression analysis for sequence count data. Genome Biology **11**(10), 106 (2010). doi:10.1186/gb-2010-11-10-r106

[7] Love, M.I., Huber, W., Anders, S.: Moderated estimation of fold change and dispersion for RNA-seq data with DESeq2. Genome Biology **15**(12), 550 (2014). doi:10.1186/s13059-014-0550-8

[8] Wang, M., Long, Q.: Modified robust variance estimator for generalized estimating equations with improved small-sample performance. Statistics in Medicine **30**(11), 1278–1291 (2011). doi:10.1002/sim.4150

[9] Liang, K.Y., Zeger, S.L.: Longitudinal data analysis using generalized linear models. Biometrika **73**(1), 13–22 (1986). doi:10.1093/biomet/73.1.13

[10] Pan, W.: On the robust variance estimator in generalised estimating equations. Biometrika **88**(3), 901–906 (2001). doi:10.1093/biomet/88.3.901

[11] Mancl, L.A., DeRouen, T.A.: A covariance estimator for GEE with improved small-sample properties. Biometrics **57**(1), 126–134 (2001). doi:10.1111/j.0006-341X.2001.00126.x

[12] Skaug, H.J.: Automatic differentiation to facilitate maximum likelihood estimation in nonlinear random effects models. Journal of Computational and Graphical Statistics **11**(2), 458–470 (2002). doi:10.1198/106186002760180617

[13] Fournier, D.A., Skaug, H.J., Ancheta, J., Ianelli, J., Magnusson, A., Maunder, M.N., Nielsen, A., Sibert, J.: AD Model Builder: Using automatic differentiation for statistical inference of highly parameterized complex nonlinear models. Optimization Methods and Software **27**(2), 233–249 (2012). doi:10.1080/10556788.2011.597854

[14] Tsonaka, R., Spitali, P.: Negative Binomial mixed models estimated with the maximum likelihood method can be used for longitudinal RNAseq data. Briefings in Bioinformatics **22**(4), 1–14 (2021). doi:10.1093/bib/bbaa264

[15] Zhang, X., Mallick, H., Tang, Z., Zhang, L., Cui, X., Benson, A.K., Yi, N.: Negative binomial mixed models for analyzing microbiome count data. BMC Bioinformatics **18**(1), 4 (2017). doi:10.1186/s12859-016-1441-7

[16] Zwiener, I., Frisch, B., Binder, H.: Transforming RNA-Seq data to improve the performance of prognostic gene signatures. PLoS ONE **9**(1), 85150 (2014). doi:10.1371/journal.pone.0085150

[17] Warton, D.I.: Why you cannot transform your way out of trouble for small counts. Biometrics **74**(1), 362–368 (2018). doi:10.1111/biom.12728

[18] Satterthwaite, F.E.: Synthesis of variance. Psychometrika **6**(5), 309–316 (1941). doi:10.1007/BF02288586

[19] Satterthwaite, F.E.: An Approximate Distribution of Estimates of Variance Components. Biometrics Bulletin **2**(6), 110 (1946). doi:10.2307/3002019

[20] Giesbrecht, F.G., Burns, J.C.: Two-Stage Analysis Based on a Mixed Model: Large-Sample Asymptotic Theory and Small-Sample Simulation Results. Biometrics **41**(2), 477 (1985). doi:10.2307/2530872

[21] Fai, A.H.T., Cornelius, P.L.: Approximate f-tests of multiple degree of freedom hypotheses in generalized least squares analyses of unbalanced split-plot experiments. Journal of Statistical Computation and Simulation **54**(4), 363–378 (1996). doi:10.1080/00949659608811740

[22] Kuznetsova, A., Brockhoff, P.B., Christensen, R.H.B.: lmerTest Package: Tests in Linear Mixed Effects Models. Journal of Statistical Software **82**(13) (2017). doi:10.18637/jss.v082.i13

[23] Nguyen, Y., Nettleton, D.: RmRNAseq: Differential expression analysis for repeated-measures RNA-seq data. Bioinformatics **36**(16), 4432–4439 (2020). doi:10.1093/bioinformatics/btaa525
